# Sex differences in brain structure: a twin study on restricted and repetitive behaviors in twin pairs with and without autism

**DOI:** 10.1186/s13229-019-0309-x

**Published:** 2019-12-31

**Authors:** Annelies van’t Westeinde, Élodie Cauvet, Roberto Toro, Ralf Kuja-Halkola, Janina Neufeld, Katell Mevel, Sven Bölte

**Affiliations:** 1 Division of Neuropsychiatry, Department of Women’s and Children’s Health, Center of Neurodevelopmental Disorders at Karolinska Institutet, Karolinska Institutet, Stockholm, Sweden; 2 Center for Psychiatry Research, Stockholm Health Care Services, Region Stockholm, Stockholm, Sweden; 3 Human Genetics and Cognitive Functions, Institut Pasteur, Paris, France; 4 CNRS UMR3571, Paris, France; 5 Université Paris Diderot, Sorbonne Paris Cité, Paris, France; 6 Department of Medical Epidemiology and Biostatistics, Karolinska Institutet, Stockholm, Sweden; 7 Laboratory for the Psychology of Child Development and Education, CNRS Unit 8240, Paris-Descartes University and Caen University, Alliance for higher education and research Sorbonne Paris Cité (IDEX), Sorbonne, France; 8 Child and Adolescent Psychiatry, Stockholm Health Care Services, Region Stockholm, Stockholm, Sweden; 9 Curtin Autism Research Group, School of Occupational Therapy, Social Work and Speech Pathology, Curtin University, Perth, WA Australia

**Keywords:** Twins, Repetitive behaviors, Neuroanatomy, Sex differences, Autism

## Abstract

**Background:**

Females with autism spectrum disorder have been reported to exhibit fewer and less severe restricted and repetitive behaviors and interests compared to males. This difference might indicate sex-specific alterations of brain networks involved in autism symptom domains, especially within cortico-striatal and sensory integration networks. This study used a well-controlled twin design to examine sex differences in brain anatomy in relation to repetitive behaviors.

**Methods:**

In 75 twin pairs (*n* = 150, 62 females, 88 males) enriched for autism spectrum disorder (*n* = 32), and other neurodevelopmental disorders (*n* = 32), we explored the association of restricted and repetitive behaviors and interests—operationalized by the Autism Diagnostic Interview-Revised (C domain) and the Social Responsiveness Scale-2 (Restricted Interests and Repetitive Behavior subscale)—with cortical volume, surface area and thickness of neocortical, sub-cortical, and cerebellar networks.

**Results:**

Co-twin control analyses revealed within-pair associations between RRBI symptoms and increased thickness of the right intraparietal sulcus and reduced volume of the right orbital gyrus in females only, even though the mean number of RRBIs did not differ between the sexes. In a sub-sample of ASD-discordant pairs, increased thickness in association with RRBIs was found exclusively in females in the orbitofrontal regions, superior frontal gyrus, and intraparietal sulcus, while in males RRBIs tended to be associated with increased volume of the bilateral pallidum.

**Limitations:**

However, due to a small sample size and the small difference in RRBI symptoms within pairs, the results of this exploratory study need to be interpreted with caution.

**Conclusions:**

Our findings suggest that structural alterations of fronto-parietal networks in association with RRBIs are found mostly in females, while striatal networks are more affected in males. These results endorse the importance of investigating sex differences in the neurobiology of autism symptoms, and indicate different etiological pathways underlying restricted and repetitive behaviors and interests in females and males.

## Background

Autism spectrum disorder (ASD) is a neurodevelopmental condition of complex origin, defined by challenges in social communication and interaction alongside restricted and repetitive behaviors and interests (RRBIs) that cause significant impairment in daily life functioning [1]. A markedly skewed sex distribution has been consistently reported in ASD, despite recently improved recognition of autism in females [2]; the ratio is still estimated around 2–3 (males):1 (females) [2, 3]. The sex bias in ASD is hypothesized to arise from a female protective effect alongside male risk factors [4]. In addition, differences might exist in the underlying etiology and symptom presentation of ASD in females, which could be associated with both reduced risk of developing ASD, as well as failure of recognizing ASD in females [5]. Thus, investigating sex differences in the neurobiology associated with ASD symptom domains is crucial to understand pathways leading to ASD in both men and women. Moreover, recent research domain criteria (RDoC) guidelines by the National Institutes of Health advise to quantify symptoms and functional domains for research purposes, rather than condensing them as categorical entities, in order to effectively investigate full variation of behaviors from typical to atypical. The latter is particularly relevant for ASD, since autistic traits have been found to be continuously distributed in the general population [6, 7].

Sex differences in symptom presentation of ASD have predominantly been reported in the domain of RRBIs. Despite some inconsistencies, see, e.g., [8], most studies have found reduced frequency and severity of RRBIs in females [9, 10], in particular less special, narrow, and intense interests [11]. These differences might be caused by divergent etiological pathways of restricted and repetitive behavior and interests (RRBIs) in autism, including underlying brain anatomy. However, so far, the brain anatomy associated with RRBIs has mainly been studied in ASD males. RRBIs have been associated with cortico-striatal circuits that connect lateral orbitofrontal, anterior cingulate cortex, and precentral motor regions to the striatum [12, 13]. In autistic males, the majority of neuroanatomical studies of RRBIs focused on subcortical areas. Here, the most conclusive finding was regional enlargement in both children and adults, in particular in the caudate nucleus [14] and the globus pallidus [15]. In addition to the cortico-striatal circuit, volume enlargements were found for the thalamus [16] and amygdala [13]. However, some reductions in volumes were observed as well, for example in the inferior frontal gyri and cerebellum [13].

Previous studies have investigated sex differences in brain structure regardless of RRBI symptom severity, and found non-overlapping structural changes in males and females [17], altered functional connectivity of the frontal lobe in males but not females [18], and sex-specific white matter connectivity [19]. However, only one study has specifically addressed sex differences in brain anatomy related to RRBIs, on the ABIDE dataset assessing 25 females and 25 males with ASD. The authors reported that gray matter of motor regions could discriminate boys from girls with ASD [20]. In addition, only in girls RRBIs were related to increased gray matter of the motor cortex, the supplementary motor area, and Crus 1 subdivision of the cerebellum, while correlating with the right putamen in boys [20]. These findings indicate a different relationship between brain anatomy and RRBIs for males and females with ASD, thus potentially pointing to divergent etiological pathways to inflexible behaviors between the sexes.

More generally, ASD is associated with environmental, shared and non-shared, as well as genetic components which likely contribute to the heterogeneity in the etiology [21, 22]. The use of a co-twin control design enables the study of neuroanatomical variation associated with RRBI symptoms independent from familial factors, i.e., genetic and environmental factors shared by twins of a pair, with 100% of genetics shared by MZ twins and on average 50% for DZ twins. This approach might enhance the sensitivity for detecting effects of non-shared environment factors. For example, life-long presence of RRBI symptoms themselves might alter brain-structure, and thus comprise a non-shared environment factor between the twins. In addition, a co-twin design reduces heterogeneity caused by age, gender, and socio-economic background. Previous twin studies have observed structural changes in brain regions relevant for RRBIs, including the caudate nucleus, pre- and postcentral gyri and cerebellum (see [23] for a review). However, none of these studies has addressed sex differences RRBI symptoms directly.

As part of the Roots of Autism and ADHD Twin Study Sweden (RATSS) [24], the objective of this explorative study was to examine sex differences in the neuroanatomy of regions of interest in relation to a dimensional estimate of RRBIs using a within-pair twin design. Surface-based estimates, including volume, surface area, and thickness of regions relevant for RRBIs were analyzed in same-sex twins aged 9 to 23 years. This sample consisted of typically developing twin pairs, in addition to pairs being concordant or discordant for ASD and other neurodevelopmental conditions.

## Methods

### Participants

Complete twin sample characteristics are presented in Table 1. Informed written consent was obtained from all participants and/or their legal guardians according to the Declaration of Helsinki. The RATSS project and the current study are approved by the regional Ethical Review Board. Twin pairs are mostly recruited from the Child and Adolescent Twin Study in Sweden (CATSS [25];) based on the Autism-Tics, ADHD, and other comorbidities inventory (ATAC) [26]. Twin pairs with at least two points difference on the ASD or ADHD subscale from the ATAC were prioritized and pairs where both scored either above or below the cut-off for ASD or ADHD were also selected. Selection was further based on aiming for a balance in sex and zygosity. Diagnosis was determined after clinical assessment in the lab. As a result of the procedure, many participants got diagnosed with other neurodevelopmental disorders in addition or in place of ASD and ADHD. However, as we were interested in the dimensional trait RRBI, we have not excluded any participants based on diagnosis. It must be noted though that we prioritized selection of discordant twin pairs. Such a selection criterion might make differences associated with the traits of interest more pronounced in our sample, especially when focusing on MZ discordant twins. This approach enables us to detect subtle associations between brain and behavior. However, concordance in the general population might be expected to be higher. As we are not aiming to estimate prevalence of concordance, nor heritability or any other quantification of gene and environment contribution, we believe that this will not pose problems to the interpretation of our results. The frequency distribution of RRBI symptoms across different diagnostic groups can be found in Table 2. In total *n* = 288 twins included in RATSS to date, *N* = 261 had completed MRI scanning, from which we only included same-sex pairs with high-quality image scans for both twins. These inclusion criteria resulted in a sample of 75 same-sex pairs (*n* = 150, age 9–23 years), of which 44 were male pairs (mean age 15.9 years) and 31 female pairs (mean age 16.4 years), and 46 monozygotic and 29 dizygotic pairs. Zygosity was determined with DNA testing (using a 48 single-nucleotide polymorphisms panel [27]) for most pairs, apart from 1 male pair who’s zygosity was established with a questionnaire, as DNA testing had not been completed yet. The sample included 32 twins with ASD (20 males, 12 females) from 20 ASD discordant (only one twin of a pair received an ASD diagnosis) and six ASD concordant pairs (both twins of a pair received ASD diagnosis), 34 twins with ADHD (23 males, 11 females), 21 twins with other neurodevelopmental disorders (13 males, eight females), and 70 without a diagnosis (40 males, 30 females). Other NDDs included mostly specific learning impairments (*n* = 13), tic disorder (*n* = 4), speech sound disorder (*n* = 2), Tourette’s disorder (*n* = 1), and language disorder (*n* = 1). Raw number of participant diagnoses are given, but participants could have more than one diagnosis.

**Table 1:** Whole twin sample and sex specific characteristics

Demographics	All (*N* = 150)	Males (*N* = 88)	Females (*N* = 62)	χ^2^, *P*
Number of pairs	75	44	31	
Age mean (*SD*)	16.10 (*3*.*31*)	15.90 (*3*.*17*)	16.39 (*3*.*51*)	.61, .435
Age range	9–23.69	10.69–23.34	9–23.69	
*N* subjects zygosity MZ/DZ	92/58	50/38	42/20	1.65, .198
*N* subjects ASD diagnosis	32	20	12	.09, .769
*N* subjects ASD diagnosis per MZ / DZ	15/17	7/13	8/4	
*N* pairs concordant for ASD	6	3	3	
*N* pairs discordant for ASD	20	14	6	
*N* pairs ASD concordant per MZ/DZ	4/2	1/2	3/0	
*N* pairs ASD discordant per MZ/DZ	7/13	5/9	2/4	
*N* subjects ADHD diagnoses	34	23	11	1.02, 0.312
*N* subjects Other neurodevelopmental disorders	25	16	9	0.14, 0.711
*N* subjects psychiatric diagnoses	24	14	10	< 0.001, 1
N subjects No diagnosis	70	40	30	0.04, 0.851
Age ASD discordant pairs, range	16.21 (3.55) 10.69–23.34	17.70 (3.16) 12.86–21.52	15.58 (3.57) 10.69–23.34	3.14, 0.076*
Age ASD concordant pairs, range	16.28 (4.08) 11.09–23.69	19.30 (3.47) 16.34–23.69	13.26(1.65) 11.09–14.43	8.43, 0.004**
Age non-ASD pairs, range	16.03(3.14) 9.00–22.77	15.63(3.37) 9.00–22.17	16.36(2.94) 10.95–22.77	1.61, 0.205

*MZ* monozygotic, *DZ* dizygotic. Concordant: both twins with ASD diagnosis, Discordant: only one sibling with ASD diagnosis. *P* values and chi-squares from test comparing males and females (χ^2^ tests for categorical, Kruskal-Wallis for numerical variables). Total number of participants for each diagnosis are displayed, but participants could have more than one diagnosis. *N* subjects gives the number of individuals, *N* pairs give the number of pairs (which includes two individuals)

**= *p* <0.01, * *p*<0.05, . = *p*< 0.1

**Table 2:** Frequency distribution of RRBI symptoms (ADI-R C) across diagnostic groups

Number of RRBIs	ASD	ADHD	Other NDD	TD	ID
0	1	13	5	66	3
1	4	4	4	9	0
2	11	3	0	3	0
3	7	1	0	3	1
4	4	1	0	0	0
5	2	1	0	1	0
6	2	0	0	0	0
10	1	0	0	0	0

*ASD* autism spectrum disorder, *ADHD* attention deficit hyperactivity disorder (but no co-morbid ASD), *Other NDD* other neurodevelopmental disorder (but no co-morbid ASD or ADHD), *TD* typically developing, *ID* intellectual disability

### Measures

#### Behavioral assessments

The comprehensive phenotypic assessment protocol of RATSS is described elsewhere in detail [24]. Briefly, clinical consensus diagnosis of ASD and other neurodevelopmental disorders or absence of clinical diagnosis was based on DSM-5 criteria [28] by three experienced clinicians, supported by information from the Autism Diagnostic Interview-Revised (ADI-R) [29], the Autism Diagnostic Observation Schedule-2 [30], the Kiddie Schedule for Affective Disorders and Schizophrenia [31], or the Diagnostic Interview for ADHD in Adults [32]. In addition, we assessed full-scale IQ (Wechsler Intelligence Scales for Children and Adults, Fourth Editions) [33, 34] and handedness (Edinburgh Handedness Inventory [35] on a scale from − 100% (left handed) to + 100% (right handed)).

The frequency and severity of RRBIs was determined by the RRBI subscale (domain C) of the ADI-R, using item codes for lifetime symptom presentation (“ever”). In the diagnostic algorithm of the ADI-R, the RRBI subscale comprises eight items scored 0 to 2, with “0” indicating no RRBIs typical of autism, “1” RRBIs typical of autism, but mild, or “2” RRBIs prototypic of autism [max. total score = 16]. The diagnostic cut-off for presence of clinically relevant RRBIs indicating ASD on the total score ≥ 2 (*n* = 41 in our sample). The ADI-R is an expert-based thorough interview reliably assessing the presence of true RRBIs by intense questioning. Therefore, a one-point difference within a twin pair on the ADI-R is quite robust. In particular since the investigation is done within a family, this interview technique has the power to get a valid contrast between twins of a pair. In our sample, 37 pairs had a within-pair difference on RRBIs of at least one point. Please, see Table 3 and Additional file 1: Figure S1, for the ADI-R RRBI score distribution in our twin sample. Further, post-hoc analyses addressed the robustness in terms of operationalization and time frame using a different RRBI estimate, the Restricted Interests and Repetitive Behavior (RRB) subscale of the Social Responsiveness Scale-2 (SRS-2) standard child or adult version [36]. The SRS-2 assesses autistic-like behaviors and quantifies its severity focusing on the past six-month, as opposed to the life-time symptom assessment of the ADI-R. Raw scores on the SRS-2-subscale autism mannerisms were retrieved as recommended for research settings [36]. The autism mannerisms subscale comprises of 12 items scored 0 to 3 on a Likert-scale [max. total score = 36], with higher scores indicating the presence of more autistic mannerisms, including repetitive behaviors and restricted interests. In our sample, 40 pairs (17 female pairs) had a within-pair difference of at least three points on the autism mannerisms subscale of the SRS-2. General cognitive abilities have been shown to affect outcome on SRS-2 raw scores, therefore IQ was corrected for in all analyses [37]. In addition, in order to test the specificity of potential brain anatomical findings to RRBI’s, against social cognition aspects of autism, we also used the social cognition subscale from the SRS-2, comprising 12 items [max. total score = 36] assessing past 6 months social cognition abilities, as well as the reciprocal interaction domain (domain A) from the ADI-R, comprising 16 items assessing life-time reciprocal interactions [max. total score = 32]. For all subscales, a higher score indicates more problems with RRBI’s, social cognition and reciprocal interaction respectively.

**Table 3:** Twin sample characteristics for the behavioral variables

	All (*N* = 150)	Males (*N* = 88)	Females (*N* = 62)	χ^2^, *P*
A) Mean scores				
IQ mean (*SD*)	97.86 (*15.65*)	97.22 (*14*.*42*)	98.77 (*17*.*31*)	.14, *.708*
Range	62–142	62–123	63–142	
ADI-R RRBI mean (*SD*)	1.02 (*1*.*62*)	1.10 (*1*.*81*)	0.90 (*1*.*33*)	.08, *.774*
Range	0–10	0–10	0–5	
ADI-R social interaction mean *(SD)*	4.08 (*5.13*)	4 (*5*.*41*)	4.19 (*4*.*76*)	.27, *0.601*
Range	0–25	0–25	0–16	
Pairs ADI-R RRBI difference^a^	37	21	16	
ASD Discordant pairs	18			
ASD Concordant pairs	8			
No-ASD pairs	15			
SRS-2 total score mean (*SD*)	41.66 (*30*.*97*)	40.65 (*29*.*50*)	43.10 (*33*.*15*)	.01, *.927*
Range	0–131	4–131	0–130	
SRS-2 RRB mean (*SD*)	5.85(*6*.*25*)	6.08 (*6*.*41*)	5.52 (*6*.*07*)	.27, *.601*
Range	0–26	0–26	0–23	
SRS-2 social cognition mean *(SD)*	7.66 (*6.50*)	7.20 (5.88)	8.31 (*7*.*29*)	.29, *.591*
Range	0–29	0–23	0–29	
ADOS-severity	2.66 (2.25) 1–10	2.91 (2.44) 1–10	2.31 (1.92) 1–8	3.07, 0.080^a^
ADOS-severity in ASD-diagnosed Participants (20 males, 12 females)	5.78 (2.52) 1–10	6.40 (2.44) 1–10	4.75 (2.42) 2–8	3.43, 0.064^a^
Handedness (mean)	66.07	66.72	65.15	1.65, *.199*
B) Within-pair differences				
IQ mean (*SD*)	10.15 (*8*.*81*)	9.84 (*9*.*48*)	10.58 (*7*.*90*)	.78, *.377*
Range	0–39	0–39	0–31	
ADI-R RRBI mean *(SD)*	1.05 (*1*.*36*)	1.022 (*1*.*36*)	1.097 (*1*.*40*)	.07, *.790*
Range	0–5	0–5	0–5	
ADI-R social interaction mean (*SD*)	2.93(4.27)	3.23 (5.27)	2.52 (3.85)	.02, *.890*
Range	0–25	0–25	0–15	
SRS-2 total score mean (*SD*)	23.05 (*26*.*22*)	23.39 (*25*.*55*)	22.58 (*27*.*57*)	< .001, .991
Range	0–101	0–96	0–101	
SRS-2 RRB mean (*SD*)	4.92 (5.77)	5.07 (5.93)	4.71 (5.62)	.05, *.827*
Range	0–22	0–22	0–22	
SRS-2 SCOG mean *(SD)*	5.4(5.35)	5.32(4.79)	5.52(6.14)	.14, *.709*
Range	0–22	0–19	0–22	

*SD* standard deviation, *IQ* overall intellectual quotient measured with WISC/WAIS. Handedness: assessed with Edinburgh handedness inventory, a scale from **−** 100/+ 100, where **−** 100 represents totally left handed, and +100 totally right handed. P-values and chi-squares are given from χ^2^ tests comparing males with females

^a^ADI-R C difference refers to the number of pairs that have a within-pair difference on the ADIR C score of > 0 and therefore contribute most to the within-pair analyses

**= *p* <0.01, * *p*<0.05, . = *p*< 0.1

### Structural MRI

#### Image acquisition

T1-weighted images were acquired on a 3 Tesla MR750 GE scanner at the Karolinska Institutet MR center (Inversion Recovery Fast Spoiled Gradient Echo - IR-FSPGR, 3D-volume, 172 sagittal slices, 256x256, FOV 24, voxel size 1 mm^3^, flip angle 12, TR/TE 8200/3.2, using a 32-channel coil array). T1-weighted acquisition was the first 7-min scan of a 50-min scanning protocol, preceded by a 5 to 7 min mock scan training for self-control of head movements. During the mock scan training participants were provided with feedback about excess head movement (1.5 mm in any direction), by automatically stopping the movie they were watching. Head movement reduced during the practice for most participants.

#### Surface-based neocortical and subcortical analyses: cortical volumetry, cortical thickness, and surface area (Freesurfer 6)

Raw images were processed in Freesurfer 6 (http://surfer.nmr.mgh.harvard.edu/). The well-established standard pipeline was run on the original T1-weighted images [38, 39]. Briefly, the intensity of the images was normalized, the brain was skull stripped, and brain tissues were segmented. A white matter volume was generated, from which a surface tessellation was created. Meshes were constructed for gray and white matter out of approximately 150,000 vertices per hemisphere, then parcellated according to the Destrieux Atlas [40]. Next, mean cortical thickness, volume, and surface areas were obtained for each region in each hemisphere. Whole brain volume from FreeSurfer was used as a covariate in all surface- and volume-based analyses except for cortical thickness, because cortical thickness is less related to brain volume [41]. After a quality control of the brain data processed from an initial 261 subjects that had completed MR scanning, 150 participants with three outputs each (cortical volume, surface area, and cortical thickness) were retained in the final surface-based analyses. Quality control was done by visual inspection of the T1 images for presence of movement errors, accuracy of skull stripping, and accuracy of the FreeSurfer segmentation, i.e., inspecting if the pial and white matter surfaces accurately followed the intersection between brain/CSF and gray matter/white matter respectively. Minor segmentation errors, such as at the temporal poles, were tolerated, in particular with regard to the young age of the subject group. Subjects were given a score on movement and image quality, of 1 (no errors)—4 (very severe movement), and only subjects with a score of 1 or 2 were included. Across pairs, age predicted movement and data quality, with younger subjects moving more (*B* = − 0.04, *p* < 0.001) and thus having less image quality (*B* = − 0.03, *p* = 0.043). However, our estimate of interest, RRBI’s (ADI-R) did not predict data quality or movement scores either across (quality: *B* = − 0.02, *p* = 0.42; movement: *B* = 0.007, *p* = 0.8) or within pairs (quality: *B* = 0.009, *p* = 0.814; movement: *B* = − 0.009, *p* = 0.889). From the subjects that were excluded due to excess movement, the mean symptom level of RRBI’s from the ADI-R C domain was 0.94, i.e., they did not have more RRBI symptoms, and the mean age of this group was slightly younger, 15.28 years, compared to 16.11 in the included sample. Finally, to assess the impact of data quality on our outcome, we ran the main analyses, i.e., the association between RRBIs from the ADI- R C domain and brain structure within pairs, split by sex, also on a subsample consisting of those with QC1 (*n* = 70), which largely replicated our findings (see Additional files).

#### Volume-based cerebellar analysis: gray and white matter regional volume (FSL)

Volumes of cerebellar white and gray matter were retrieved using volume-based morphometry. The 261 raw brain volumes were intensity normalized and the brain was extracted using AFNI’s 3dskullstrip. Skull stripped 3D images were segmented in 3 tissue types (Gray Matter-GM, White Matter-WM, Cerebral Spinal Fluid-CSF) using FAST (FMRIB's Automated Segmentation Tool within FMRIB's Software Library) which also corrects for spatial intensity variation. Segmented images were warped to MNI space using non-linear registration FNIRT from FSL. GM and WM volumes for the somato-motor cerebellar region were extracted from the intersection between the somato-motor regions in Buckner’s 7-network functional atlas, which includes anatomical regions IV, V, VI, and VIIB of the cerebellum [42], and segmented individual volumes using a custom script in C. The same 150 individuals that had passed the surface-based quality control were included in the volume-based analyses. These 150 scans all had good segmentation quality in FSL.

#### ROI selection for the neocortical, subcortical and cerebellar RRBI networks

RRBIs are presumed to rely on a wide network of regions involved in motor function and cognitive control of neocortical and subcortical areas, in particular cortico-striatal circuits [43]. In the current study, we therefore focus on these cortico-striatal loops, motor regions, and sensory integration areas, that have previously been associated with ASD, including pre- and post-central motor regions, the striatum [12], the amygdala [13], and sensory-motor integration areas in the posterior parietal cortex [44], and areas involved in executive functioning in prefrontal areas [12] and cerebellum [45]. Based on these previous findings, we selected a priori corresponding neocortical and subcortical regions of interest within the Destrieux atlas from Freesurfer [40]. We included volumes, surface area, and thickness of 18 bilateral regions, namely the anterior cingulate cortex (ACC), lateral orbital sulcus, orbital gyrus, inferior frontal orbital gyrus, postcentral gyrus, postcentral sulcus, precentral gyrus, precentral inferior sulcus, precentral superior sulcus, central sulcus, superior frontal sulcus, superior frontal gyrus, middle frontal sulcus, middle frontal gyrus, supramarginal gyrus, superior parietal lobule, intra parietal sulcus and angular gyrus, as well as volumes of five subcortical regions, namely the bilateral caudate nucleus, globus pallidus, putamen, thalamus, and amygdala, in addition to volume of the cerebellar cortex and white matter. We also included the volume of the somato-motor region of the cerebellum based on a functional connectivity atlas from FSL [42].

### Statistical analysis

All statistical analyses were performed in R (https://www.**r**-project.org/).

#### Sex differences in demographics

We first examined possibly confounding demographic differences between females and males. Statistical comparisons between the sexes were conducted using χ^2^ tests for categorical variables (zygosity, diagnosis) and Kruskal-Wallis tests for continuous variables (age, RRBIs, IQ, handedness scores). Some of the variables were not normally distributed; for consistency reasons, non-parametric tests were chosen for all tests. These tests yielded no between group significant differences (see Table 1).

#### Twin/co-twin: within-pair differences in RRBIs associated with within-pair differences in neuroanatomy of the motor network

The main analyses focused on within-pair differences in RRBIs as assessed with the ADI-R C domain, while post-hoc control analyses (1) cross-validated the findings with the RRB subscale from SRS-2 and (2) tested the specificity of the findings to RRBIs by controlling for social cognition. Total brain volume was adjusted for when assessing cortical volume and surface area, but not thickness, and IQ was adjusted for in all models.

For the main analyses, a twin/co-twin design was implemented to investigate the association between RRBI’s on a dimensional scale (predictor) and the anatomy (outcome) of the regions of interest while controlling for unmeasured confounding factors shared within twin pairs (e.g., genetic factors, demographics etc.). MZ and DZ twins were collapsed in order to increase statistical power. Within-twin pair associations were estimated using a conditional linear regression model within the generalized estimating equations (GEE) framework, using the dergee package from R [46]. Herein, the difference in the exposure variable within a pair is correlated to the difference in the outcome variable within the same pair, thus yielding an estimate of a within-pair association (see Additional file 1: Figure S2 and Figure S3 for some examples). This within-pair relationship is calculated for all pairs, resulting in an estimate for the average within-pair association between RRBIs and brain anatomy in the group. This association was thus estimated by using dimensional differences within-twin pairs, i.e., differences within pairs on total scored points of RRBIs.

##### Main within pair effects of RRBIs (ADI-R) on brain anatomy for males and females

Within-pair analyses were run in three sub-steps. First, the association between RRBIs and brain structure was assessed for males and females separately.

##### Sex-specific regional alterations

Next, to compare the association between symptoms and structure in males and females, we computed Wald χ^2^ tests for each ROI that was associated with RRBIs in either males or females. A significant difference on a Wald-test indicates that the estimate of the association was different for the sexes. By doing the interaction analysis in this way, we could allow for confounding covariates to differ between sexes.

##### Sex-specific results: testing robustness and specificity of the effects

Further, to test the robustness of the observed effects, models otherwise identical to the models of the main analyses were run with a different estimate of RRBIs, RRB subscale of the SRS-2, which addresses current as opposed to life-time symptoms. Finally, specificity of the results toward RRBIs were tested by adding different autism symptom domains as covariates in the model, including the social cognition subscale from SRS-2, and reciprocal social interaction domain from the ADI-R, to control for highly correlated symptoms that might have confounded the observed effects. Additional analyses were run to control for interaction effects between age and RRBIs on brain anatomy showing significant associations for the right postcentral gyrus, superior precentral sulcus, and superior parietal sulcus, i.e., areas which were not associated with RRBIs in our study (Additional file 2: Table S10A and B). Further, we re-ran our analyses on a subsample of participants that were either concordant (*n* = 6 pairs) or discordant (*n* = 20 pairs) for ASD diagnosis (Additional files).

#### Multiple Comparisons Correction and Power

All *p* values of the brain–RRB symptom associations are FDR corrected for type I errors, significance threshold was set to *q* < .05. However, we also report results with *q* < 0.1 in order to not miss potentially relevant, but sub-threshold findings. FDR-corrections were performed per sub-test. For example, FDR was performed on all *p* values from the comparison: cortical thickness of 36 regions (18*2 hemispheres) in males associated with RRBIs. A separate FDR-correction was performed on all *p* values for the same comparison but in females. It must be noted that the model included sex as a factor, that is, only one model was run including both sexes, even though a *p* value list of outcomes for each sex was generated, on which the FDR correction was performed. The total number of comparisons in the main analyses is 36 (18*2) cortical regions * 3 estimates (thickness, area, volume) + 10 subcortical regions (volume) + 6 cerebellar regions (volume of bilateral grey, white and somatomotor grey, and white) = 124 associations per sex. Post-hoc Wald-tests were performed in order to compare males to females only for those regions that were significantly associated with brain structure in either males or females. Therefore, no multiple comparison correction was conducted on these tests. Further post-hoc analyses that were preformed included 124 comparisons each per sex for associations between brain structural estimates with the RRB sub-scale of the SRS-2; the ADI-R sub-scores A (Social Interaction) and C (RRBI); the SRS-2 subscales RRB and Social cognition, and finally the interaction between RRBI’s from ADIR and age. FDR correction was performed per estimate (thickness, area, volume) per test. In addition, we calculated sex differences between demographic data, with a total number of six tests. Across and within subjects’ associations between the different variables also included six tests each. The behavior associations and sex difference calculations were descriptive in nature. Therefore, no multiple comparisons corrections were performed. Sample size of the present study was comparable to recently published twin studies using similar co-twin designs reporting medium to large effect size [47, 48].. At the same time, sex-differences in grey matter volume after correcting for total brain volume are expected to be small [49].

## Results

### Sex differences in demographics

Males and females did not differ on overall RRBI symptom severity, other autistic symptoms and traits and IQ. Further, no within pair differences between the sexes were observed for any of these variables (Table 3).

### Twin/co-twin: within-pair differences in RRBIs associated with within-pair differences in neuroanatomy of the motor network

#### Main within pair effects of RRBIs (ADI-R) on brain anatomy for males and females

**Fig. 1:**
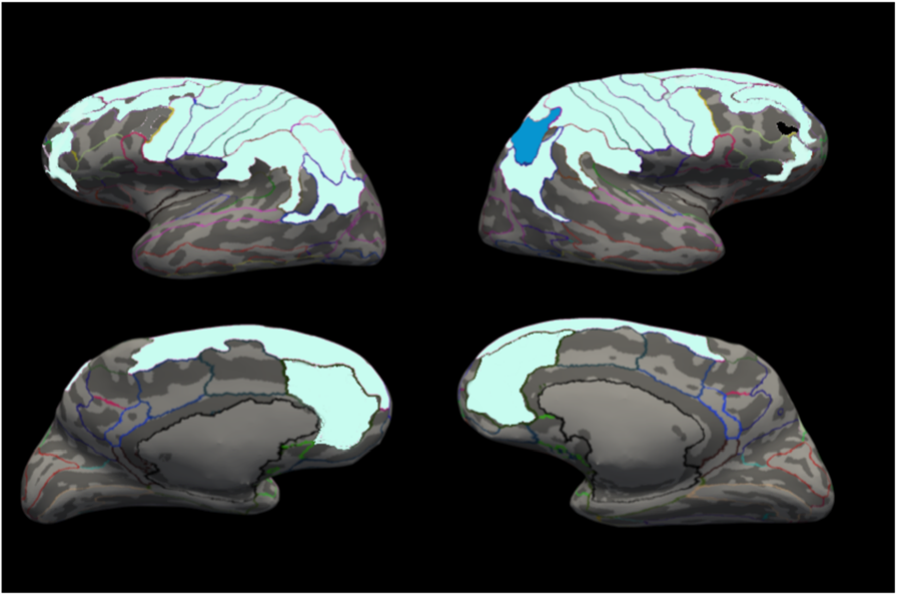
Brain region associated with restricted and repetitive behaviors and interests in females. Within-pair association between ADI-R C and brain structure. The area that was significantly associated with RRBIs is displayed blue: increased thickness of the right intraparietal sulcus in females. Areas not significantly associated with RRBIs, but included in our regions of interest, are displayed in soft green

Main results are presented in Tables 4 and 5. When splitting the sample by sex and controlling for IQ, within-pair increases in RRBI symptoms were related to increased thickness of the right intraparietal sulcus in females only (*B* = 0.037, *q* = 0.012) (see Fig. 1 and Additional file 1: Figure S3). No other significant associations were observed. However, reduced surface area in the same region was found at *q* < 0.1 (*B* = 120.61, *q* = 0.072). Moreover, there were associations only at *q* < 0.1 in females between RRBI symptoms and increased thickness of the right orbital gyrus (*B* = 0.05, *q* = 0.056) and right inferior frontal orbital gyrus (*B* = 0.07, *q* = 0.065), and reduced surface area of the left superior frontal gyrus (*B* = − 130.44, *q* = 0.072). Increased surface area of the right middle frontal gyrus in relation to more RRBIs was observed when using a threshold of *q* < 0.1 (*B* = 95.29, *q* = 0.072). In males, on the other hand, no within-pair associations between RRBIs and brain anatomy were observed at any threshold. We did not observe any significant associations between subcortical or cerebellar regions and RRBIs in either sex.

**Table 4:** Twin model associations between cortical volume, surface area and thickness of neocortical regions of interest (ROIs) and RRBI symptoms

ROIs Neocortical	Cortical volume		Surface area		Cortical thickness	
	Males β (SD) *q*	Females β (SD) *q*	Males β (SD) *q*	Females β (SD) *q*	Males β (SD) *q*	Females β (SD) *q*
Right lateral orbital sulcus	**−** 25.19	21.64	**−** 0.78	0.95	**−** 0.03	0.02
	(18.5)	(17.93)	(8.86)	(9.59)	(0.016)	(0.02)
	*0.889*	*0.554*	*0.971*	*0.976*	*0.752*	*0.667*
Left lateral orbital sulcus	**−** 10.35	47.58	**−** 6.75	2.47	0.00	0.06
	(13.23)	(24.76)	(6.79)	(8.49)	(0.02)	(0.04)
	*0.895*	*0.246*	*0.971*	*0.976*	*0.999*	*0.42*
Right middle frontal sulcus	**−** 62.16	**−** 5.56	**−** 23.66	5.97	0.00	**−** 0.003
	(75.19)	(93.0)	(20.28)	(30.69)	(0.02)	(0.02)
	*0.895*	*0.962*	*0.971*	*0.976*	*0.999*	*0.91*
Left middle frontal sulcus	**−** 46.91	27.27	**−** 3.27	3.45	**−** 0.01	**−** 0.01
	(70.37)	(64.08)	(22.93)	(18.61)	(0.02)	(0.02)
	*0.895*	*0.894*	*0.971*	*0.976*	*0.924*	*0.715*
Right orbital gyrus	19.63	138.56	7.07	9.8	**−** 0.01	**0.05**
	(57.06)	(49.29)	(15.3)	(16.91)	(0.01)	**(0.02)**
	*0.931*	*0.178*	*0.971*	*0.976*	*0.923*	***0.056***
Left orbital gyrus	97.56	**−** 3.72	11.84	**−** 7.86	0.004	0.019
	(62.59)	(77.79)	(13.27)	(15.18)	(0.02)	(0.02)
	*0.714*	*0.962*	*0.971*	*0.976*	*0.984*	*0.667*
Right middle frontal gyrus	83.36	285.63	10.68	**95.29**	0.01	**−** 0.02
	(138.41)	(143.27)	(35.28)	**(34.68)**	(0.009)	(0.02)
	*0.895*	*0.246*	*0.971*	***0.072***	*0.924*	*0.608*
Left middle frontal gyrus	**−** 11.56	87.38	18.94	13.73	**−** 0.01	**−** 0.001
	(160.84)	(173.55)	(35.76)	(36.58)	(0.01)	(0.02)
	*0.944*	*0.854*	*0.971*	*0.976*	*0.923*	*0.955*
Right inferior frontal orbital gyrus	61.59	**−** 25.2	10.41	**−** 10	0.02	**0.07**
	(22.08)	(36.49)	(4.38)	(7.67)	(0.03)	**(0.02)**
	*0.190*	*0.840*	*0.564*	*0.769*	*0.924*	***0.065***
Left inferior frontal orbital gyrus	6.15	30.12	1.05	0.84	0.01	0.02
	(19.3)	(28.32)	(4.38)	(5.88)	(0.02)	(0.02)
	*0.931*	*0.609*	*0.971*	*0.976*	*0.924*	*0.667*
Right anterior cingulate cortex	3.65	**−** 45.16	**−** 14.55	**−** 8.86	0.007	0.002
	(51.55)	(90.27)	(24.12)	(26.81)	(0.02)	(0.01)
	*0.944*	*0.854*	*0.971*	*0.976*	*0.924*	*0.910*
Left anterior cingulate cortex	74.36	64.47	15.22	5.2	0.005	0.019
	(81.02)	(59.73)	(27.96)	(16.14)	(0.01)	(0.01)
	*0.895*	*0.609*	*0.971*	*0.976*	*0.926*	*0.416*
Right postcentral gyrus	98.99	95.24	13.86	24.14	0.017	0.01
	(85.47)	(51.44)	(23.53)	(19.86)	(0.02)	(0.02)
	*0.889*	*0.256*	*0.971*	*0.778*	*0.923*	*0.715*
Left postcentral gyrus	53.83	**−** 20.69	25.34	**−** 1.88	0.007	0.005
	(61.86)	(93.6)	(25.32)	(34.76)	(0.02)	(0.02)
	*0.895*	*0.900*	*0.971*	*0.976*	*0.926*	*0.828*
Right postcentral sulcus	**−** 143.93	52.32	**−** 64.7	9.43	0.006	0.02
	(90.46)	(66.92)	(36.76)	(29.48)	(0.01)	(0.008)
	*0.714*	*0.782*	*0.933*	*0.976*	*0.924*	*0.14*
Left post central sulcus	58.03	**−** 71.79	17.06	**−** 27.95	0.003	0.015
	(86.65)	(50.89)	(41.72)	(25.47)	(0.01)	(0.01)
	*0.895*	*0.525*	*0.971*	*0.778*	*0.984*	*0.416*
Right precentral gyrus	**−** 45.01	**−** 22.53	**−** 7.78	20.51	**−** 0.003	**−** 0.02
	(82.87)	(71.65)	(24.97)	(18.62)	(0.02)	(0.019)
	*0.900*	*0.900*	*0.971*	*0.778*	*0.984*	*0.482*
Left precentral gyrus	**−** 78.17	62.85	5.9	32.14	**−** 0.011	**−** 0.019
	(108)	(75.17)	(18.81)	(21.36)	(0.02)	(0.01)
	*0.895*	*0.764*	*0.971*	*0.596*	*0.924*	*0.143*
Right inferior precentral sulcus	**−** 38.95	**−** 4.84	**−** 26.55	1.94	**−** 0.006	**−** 0.010
	(51.56)	(51.05)	(20.55)	(19.71)	(0.017)	(0.016)
	*0.895*	*0.962*	*0.971*	*0.976*	*0.926*	*0.715*
Left inferior precentral sulcus	26.8	**−** 13.62	**−** 0.84	**−** 9.55	**−** 0.002	0.013
	(54.84)	(54.21)	(16)	(19.95)	(0.012)	(0.01)
	*0.900*	*0.900*	*0.971*	*0.976*	*0.984*	*0.550*
Right superior precentral sulcus	49.29	**−** 23.56	10.74	0.85	0.014	**−** 0.021
	(40.37)	(62.97)	(16.76)	(28.51)	(0.01)	(0.014)
	*0.886*	*0.900*	*0.971*	*0.976*	*0.923*	*0.453*
Left superior precentral sulcus	12.3	62.67	13.56	15.89	0.0002	0.015
	(44.33)	(46.71)	(20.63)	(19.52)	(0.010)	(0.011)
	*0.938*	*0.525*	*0.971*	*0.935*	*0.999*	*0.482*
Right central sulcus	17.46	59.85	9	29.72	**−** 0.006	**−** 0.008
	(73.4)	(44.51)	(32.77)	(18.95)	(0.007)	(0.007)
	*0.942*	*0.525*	*0.971*	*0.596*	*0.923*	*0.607*
Left central sulcus	5.45	38.7	**−** 5.28	16.56	**−** 0.001	**−** 0.005
	(37.96)	(64.97)	(17.64)	(33.12)	(0.009)	(0.008)
	*0.942*	*0.854*	*0.971*	*0.976*	*0.988*	*0.715*
Right superior frontal sulcus	**−** 27.93	269.97	**−** 21.4	81.83	0.018	0.011
	(146.59)	(129.92)	(45.76)	(37.33)	(0.009)	(0.014)
	*0.942*	*0.246*	*0.971*	*0.256*	*0.752*	*0.667*
Left superior frontal sulcus	14.56	108.86	**−** 11.46	30.68	0.012	0.005
	(104.96)	(90.84)	(36.81)	(28.44)	(0.009)	(0.012)
	*0.942*	*0.554*	*0.971*	*0.778*	*0.923*	*0.746*
Right superior frontal gyrus	63.05	237.18	**−** 5.09	66.53	0.011	0.007
	(158.88)	(121.69)	(45.05)	(34.86)	(0.011)	(0.009)
	*0.931*	*0.246*	*0.971*	*0.406*	*0.923*	*0.698*
Left superior frontal gyrus	**−** 278.67	**−** 363.82	**−** 96.72	**− 130.44**	0.017	0.028
	(156.8)	(187.75)	(44.93)	**(47.07)**	(0.009)	(0.012)
	*0.714*	*0.246*	*0.564*	***0.072***	*0.752*	*0.143*
Right supramarginal gyrus	134.4	21.49	**−** 3.27	15.28	0.030	0.012
	(107.4)	(93.84)	(23.98)	(35.15)	(0.026)	(0.025)
	*0.889*	*0.900*	*0.971*	*0.976*	*0.923*	*0.746*
Left supramarginal gyrus	**−** 152.85	**−** 248.39	**−** 34.84	**−** 53.37	**−** 0.007	**−** 0.012
	(97.63)	(119.75)	(39)	(35.18)	(0.015)	(0.019)
	*0.714*	*0.246*	*0.971*	*0.596*	*0.924*	*0.715*
Right superior parietal gyrus	**−** 52.18	**−** 62.3	7.1	**−** 24.15	**−** 0.01541	0.011
	(103.56)	(111.09)	(29.1)	(33.94)	(0.01462)	(0.011)
	*0.900*	*0.8541*	*0.9709*	*0.9763*	*0.9228*	*0.6287*
Left superior parietal gyrus	**−** 58.84	53.91	36.09	1.72	**−** 0.024	**−** 0.002
	(93.96)	(201.92)	(31.37)	(52.12)	(0.025)	(0.021)
	*0.8951*	*0.900*	*0.971*	*0.976*	*0.923*	*0.942*
Right intraparietal sulcus	38.48	**−** 169.78	15.22	**− 120.61**	0.011	**0.037**
	(111.05)	(88.27)	(53.82)	**(39.45)**	(0.011)	(**0**.**010**)
	*0.931*	*0.246*	*0.971*	***0.072***	*0.923*	***0****.****012***
Left intraparietal sulcus	**−** 51.43	97.68	**−** 26.31	25.49	0.008	0.018
	(80.57)	(95.6)	(36.84)	(47.17)	(0.009)	(0.011)
	*0.895*	*0.614*	*0.971*	*0.976*	*0.923*	*0.416*
Right angular gyrus	**−** 284.76	150.72	**−** 61.86	**−** 32.72	**−** 0.020	0.055
	(150.54)	(114.9)	(38.01)	(38.6)	(0.023)	(0.028)
	*0.714*	*0.525*	*0.933*	*0.935*	*0.923*	*0.244*
Left angular gyrus	**−** 113.07	71.55	**−** 1.29	25.98	**−** 0.019	**−** 0.027
	(146.84)	(128.96)	(35.37)	(29.82)	(0.029)	(0.028)
	*0.895*	*0.854*	*0.971*	*0.935*	*0.924*	*0.658*

Estimates (B), standard deviations (SD) and *q* values (*q*) for the within-pair associations between all surface-based neocortical measures (volume, surface area, and thickness) and repetitive behavior symptoms, as assessed by the ADI-R C. A positive estimate indicates an increase in brain estimate related to more repetitive behavior symptoms. Bold text indicates significant associations (FDR-corrected *q* value < 0.05), while bold and grey text indicate associations with *q* value < 0.10 (FDR-corrected)

**Table 5:** Twin model associations between subcortical volumes of subcortical regions of interest (ROIs) and RRBI symptoms

ROIs subcortical	Volume males β (SD) *q*	Volume females β (SD) *q*	ROIs cerebellum	Volume males β (SD) *q*	Volume females β (SD) *q*
Left thalamus proper	64.88	**−** 27.35	Left cerebellum white	4.42	**−** 64.51
	(58.12)	(48.61)		(160.07)	(110.1)
	*0.661*	*0.738*		*0.978*	*0.5587*
Left caudate	9.08	**−** 55.08	Left cerebellum cortex	**−** 719.02	**−** 256.43
	(40.75)	(40.32)		447.	(283.8)
	*0.915*	*0.738*		*0.215*	*0.559*
Left putamen	**−** 13.98	**−** 13.37	Right cerebellum white	9.13	**−** 85.27
	(35.49)	(25.49)		(101.93)	(102.54)
	*0.915*	*0.738*		*0.978*	*0.559*
Left pallidum	19.66	**−** 5.64	Right cerebellum cortex	**−** 664.7	**−** 282.66
	(9.52)	(12.84)		(294.83)	(341.24)
	*0.194*	*0.738*		*0.105*	*0.559*
Left amygdala	0.07	8.96	Somato motor	18.74	7.23
	(9.5)	(24.15)		(8.89)	(12.37)
	*0.994*	*0.738*		*0.105*	*0.559*
Right thalamus proper	25.28	**−** 31.66	Somato motor white	11.76	8.33
	(37.31)	(32.66)		(10.92)	(12.29)
	*0.830*	*0.738*		*0.422*	*0.559*
Right caudate	**−** 37.03	**−** 36.45			
	(47.76)	(39.31)			
	*0.830*	*0.738*			
Right putamen	62.72	**−** 23.84			
	(45.87)	(22.52)			
	*0.572*	*0.738*			
Right pallidum	29.47	**−** 6.45			
	(13.06)	(19.29)			
	*0.194*	*0.738*			
Right amygdala	4.99	**−** 13.25			
	(19.11)	(16.92)			
	*0.915*	*0.738*			

Estimates (B), standard deviations (SD), and *q* values (*q*) for the within-pair associations between all surface-based subcortical volumes and repetitive behavior symptoms, as assessed by the ADI-R C. For the whole group associations, sex was added as a covariate. A positive estimate indicates an increase in brain estimate related to more repetitive behavior symptoms. Bold text indicates significant associations (FDR-corrected *q* value < 0.05), while bold and grey text indicate associations with *q* value < 0.10 (FDR-corrected)

Moreover, when controlling for non-RRBI autism symptoms and traits on the ADI-R, to test the specificity of the observed associations in females, increased thickness of the right intraparietal sulcus was still significantly associated with RRBIs on ADI-R (*B* = 0.041, *q* = 0.008). In addition, in females, increased thickness of the right postcentral sulcus (*B* = 0.026, *q* = 0.031), and increased volume of the right orbital (*B* = 161.09, *q* = 0.007) and postcentral gyri (*B* = 154.48, *q* = 0.003) was associated with more RRBI symptoms, while controlling for other autism symptoms. For males, RRBIs were associated with reduced volume of the right cerebellum cortex (*B* = − 1092.29, *q* = 0.014) (Additional file 2: Table S8A and B). Please see Table 6 for a comparison between the main results from the ADI-R C and the outcomes when controlling for other autism symptoms.

**Table 6:** Comparison of results between the main (RRBI’s from the ADI-R C domain) and additional analyses: SRS-2 Autism Mannerisms (AM); ADI-R C + ADI-R reciprocal social interaction (A); and SRS-2 AM + SRS – social cognition (SC)). Significant B-estimates and *q* values are displayed in bold (*q* < 0.05) and estimates with a value of *q* < 0.1 are displayed as regular text. Results are FDR-corrected

Group (n)	Regions associated with RRBI’s on the ADI-R C domain & SRS-2 AM, and when correcting for social cognition symptoms						
Males ADI-R C (88)	Nothing						
Males ADIR-C + A	Bilateral cerebellum C. Decreased volume (Right: **B** = **− 1092.29,** ***q*** **= 0**.**014**; Left: *B* = **−** 1198.49, *q* = 0.070)	R. Inferior frontal G. Increased volume (*B* = 81.99, *q* = 0.093)					
Males SRS-2 AM	R. Pallidum Increased volume (***B*** = **5.99**, ***q*** = **0**.**005**)	R. Postcentral S. Reduced volume (*B* = **−** 48.93, *q* = 0.059) Reduced area (*B* = **−** 21.74, *q* = 0.060)					
Males SRS-2 AM + SC	R. Precentral G. Reduced area (*B* = **−** 27.92, *q* = 0.002)	R. Central G. Reduced area (*B* = **−** 32.08, *q* = 0.032)					
Females ADI-R C (62)	R. Intraparietal S. Increased thickness (***B*** = **0**.**037**, ***q*** = **0**.**012**) Reduced area (*B* = **−** 120.61, *q* = 0.072)	R. Orbital G. Increased thickness (*B* = 0.05, *q* = 0.056)	R. Inferior Frontal Orbital G. Increased thickness (*B* = 0.07, *q* = 0.065)	L. Superior Frontal G. Reduced area (*B* = **−** 130.44, *q* = 0.072)	R. Middle Frontal G. Increased area (*B* = 95.29, *q* = 0.072)		
Females ADIR-C + A	R. Intraparietal S. Increased thickness (***B*** = **0**.**041**, ***q*** = **0**.**008)** Reduced area (*B* = **−** 115.97, *q* = 0.075)	R. Orbital G. Increased volume (***B*** = **161**.**09**, ***q*** = **0**.**007)**	R. Inferior Frontal Orbital G. Increased thickness (*B* = 0.075, *q* = 0.055)	R. Postcentral G. Increased volume (***B*** = **154**.**48**, ***q*** = **0**.**003**)	R. Postcentral S. Increased thickness **(*B*** = **0**.**026**, ***q*** = **0**.**031)**		
Females SRS-2 AM (62)	L. Intraparietal S. Increased thickness (***B*** = **0**.**006**, ***q*** = **0**.**049)**	R. Orbital G. Increased thickness (***B*** = **0**.**013**, ***q*** = **0**.**008**)	L. Lateral Orbital S. Increased thickness (***B*** = **0**.**017**, ***q*** = **0**.**007**)	R. Supramarginal G. Increased area (*B* = 18.69, *q* = 0.007)			
Females SRS-2 AM + SC	L. Angular G. Increased thickness (***B*** = **0**.**023**, ***q*** = **0**.**018**) Bi. Angular G. Reduced area (Left: *B* = **−** 33.04, *q* = 0.054; right: *B* = **−** 31.05, *q* = 0.051)	L. Lateral orbital S. Reduced area (***B*** = **− 8.13**, ***q*** = **0**.**003)**	Bi. Middle frontal S. Reduced thickness (Left: *B* = **−** 0.009, *q* = 0.071; right: *B* = **−** 0.011, *q* = 0.067)	R. Supramarginal G. Increased area (*B* = 33.04, *q* = 0.063)	R. Superior parietal G. Increased area (*B* = 26.44, *q* = 0.066)	R. Superior frontal G. Reduced thickness (*B* = **−** 0.009, *q* = 0.055)	R. Postcentral G. Reduced thickness (*B* = **−** 0.017, *q* = 0.056)

*L* left, *R* right, *Bi* bilateral, *G* gyrus, *S* sulcus, *C* cortex

#### Sex-specific regional alterations

Further, the relationship between RRBIs on the ADI-R and brain structure differed significantly for males and females on both thickness (χ^2^ = 4.55, *p* = 0.033) and surface area (χ^2^ = 4.02, *p* = 0.045) of the right intraparietal sulcus, and of thickness of the right orbital gyrus (χ^2^ = 4.46, *p* = 0.035).

#### Sex-specific results: testing robustness and specificity of the effects

Table 6 compares the significant and sub-threshold findings between the ADI-R C, SRS-2 AM, and the analyses with social cognition as a covariate. In females, within-pair increases in current RRBIs, as assessed by SRS-2 RRB subscale, were associated with within-pair increases in thickness of the left intraparietal (*B* = 0.006, *p* = 0.049) and lateral orbital sulci (*B* = 0.017, *p* = 0.007) as well as right orbital gyrus (*B* = 0.013, *p* = 0.008), and increased surface area of the right supramarginal gyrus (*B* = 18.69, *p* = 0.007). In males, within-pair increases in current RRBIs, were associated with increased volume of the right pallidum (*B* = 5.99, *p* = 0.005). In addition, in males we observed associations when setting the threshold to *q* < 0.1, which included within-pair reduction of volume (*B* = − 48.93, *p* = 0.059) and surface area (*B* = − 21.74, *p* = 0.060) of the right postcentral sulcus (Additional file 2: Table S7A and B). However, when controlling for current social cognition impairments, these specific associations were no longer present, but other relationships did emerge in both sexes (Additional file 2: Table S9A and B).

Table 7 compares the significant and sub-threshold findings of both the main and the additional analyses on the ASD subset and the high-quality data. In a subset of ASD discordant and concordant pairs, the finding of the right intraparietal sulcus was replicated. In addition, in females there was also an association between RRBIs and increased thickness of the left lateral orbital sulcus, right orbital gyrus, and left superior frontal gyrus, while in males there was only an association between RRBIs and bilateral pallidum volume, the latter being significant only at *q* < 0.1 (Additional file). Finally, we tested the robustness of our findings in a sub-sample with very high data quality (*n* = 70). These analyses largely replicated our initial findings, but showed additional associations between cortical structure and RRBIs in females, in particular in the prefrontal cortex, but also a few associations between RRBIs and brain structure in males: the left lateral orbital sulcus (reduced surface area), ACC (increased thickness), and supramarginal gyrus (increased surface area and volume) (Additional file).

**Table 7:** Comparison of results between the main (all subjects) and additional analyses (ASD- discordant and concordant group, and the highest data quality group (High Q)). Significant B-estimates and *q* values are displayed in bold (*q* < 0.05) and estimates with a value of *q* < 0.1 are displayed as regular text. Results are FDR-corrected

Group (n)	Regions associated with RRBI’s on the ADI-R C domain						
Males Main (88)	Nothing						
Males ASD Disc (28) ASD Conc (6)	Bilateral Pallidum Increased volume (Left: *B* = 23.19, *q* = 0.096; right: *B* = 31.51, *q* = 0.096)						
Males High Q (34)	R. Supramarginal G. Increased area (***B*** = **130**.**6**, ***q*** = **0**.**035**) Increased Volume (***B*** = **842**.**22**, ***q*** = **0**.**018**)	L. Lateral orbital S. Reduced area (***B*** = − **28**.**44**, ***q*** = **0**.**035**)	L. Anterior cingulate C. Increased thickness (***B*** = **0**.**093**, ***q*** = **0**.**001**)				
Females Main (62)	R. Intraparietal S. Increased thickness (***B*** = **0**.**037**, ***q*** = **0**.**012**) Reduced area (*B* = − 120.61, *q* = 0.072)	R. Orbital G. Increased thickness (*B* = 0.05, *q* = 0.056)	R. Inferior Frontal Orbital G. Increased thickness (*B* = 0.07, *q* = 0.065)	L. Superior Frontal G. Reduced area (*B* = − 130.44, *q* = 0.072)	R. Middle Frontal G. Increased area (*B* = 95.29, *q* = 0.072)		
Females ASD disc (12) ASD Conc (6)	R. Intraparietal S. Increased thickness (***B*** = **0**.**032**, ***q*** = **0**.**013**)	R. Orbital G. Increased thickness ***B*** = **0**.**070**, ***q*** = **0**.**016**) Increased volume (*B* = 166.73, *q* = 0.059)	L. Lateral Orbital S. Increased thickness (***B*** = **0**.**110**, ***q*** = **0**.**002**)	L. Superior Frontal G. Increased thickness (***B*** = **0**.**043**, ***q*** = **0**.**003**)	R. Postcentral G. Increased thickness (*B* = 0.039, *q* = 0.087)	L. Postcentral S. Reduced volume (*B* = − 208.12, *q* = 0.059)	
Females High Q (36)	Bi. Intraparietal S. Increased thickness (Left: ***B*** = **0**.**045**, ***q*** < **0**.**001**; right: ***B*** = **0**.**053**, ***q*** = **0**.**027**)	L. Lateral orbital S. Increased thickness (***B*** = **0**.**094**, ***q*** = **0**.**002**)	L. Inferior frontal orbital G. Increased thickness (***B*** = **0**.**069**, ***q*** = **0**.**022**) Increased volume (*B* = 88.10, *q* = 0.059)	L. Superior frontal G. Increased thickness (***B*** = **0**.**059**, ***q*** < **0**.**001**) Reduced area (***B*** = − **192**.**15**, ***q*** = **0**.**010**)	L. Middle frontal G. Increased thickness (***B*** = **0**.**036**, ***q*** = **0**.**031**)	R. Postcentral G. Increased thickness (***B*** = **0**.**038**, ***q*** = **0**.**036**) Increased volume (*B* = 235.05, *q* = 0.053)	R. Postcentral S. Increased thickness (***B*** = **0**.**038**, ***q*** < **0**.**001**)

*L* left, *R* right, *Bi* bilateral, *G* gyrus, *S* sulcus, *C* cortex

## Discussion

The present twin-study is the first to assess sex differences in the anatomy of brain networks associated with RRBI symptoms in autism. Significant associations were observed mostly within female pairs with largely varying frequencies and severities of RRBI symptoms and traits. In particular, the female twin with more RRBI symptoms had increased thickness of the right intraparietal sulcus. Additional alterations were found in the orbito-frontal areas, albeit without reaching statistical significance. Despite comparable within-pair differences in RRBIs, and comparable level of total autistic impairments, such associations with brains structure were not observed in males. Our results therefore suggest that, when controlling for many shared factors between twins, associations between RRBI symptoms and brain structure are mostly found in females, and involve in particular increased thickness of the cortex.

Our observations correspond partly to the previous study on sex differences in the neuroanatomy of the motor networks in ASD [20], where brain structure of motor areas, including the motor cortex and supplementary motor area, as well as Crus 1 of the cerebellum, predicted RRBIs only in girls, while RRBIs in boys were predicted by volume of the right putamen. Further, in that study, gray matter structure of motor regions was able to distinguish boys from girls with ASD.

Thus, in addition to our study, these findings suggest mainly brain structural associations with RRBIs in females and not in males. Indeed, in our study there was a striking contrast between finding increased thickness in fronto-parietal regions in females, while there were hardly any associations between brain structure and RRBI’s in males. While Supekar and Menon report mostly primary motor regions, we observe sex-specific associations with RRBIs in females in a region involved in visuo-motor coordination and intention interpretation (intra-parietal sulcus), that has also been shown to be involved in shifting attention and motor learning [50], in addition to a non-significant association (*q* < 0.1) in a region involved in executive function and decision making (orbital gyri) [51, 52]. These findings correspond to the hypothesis that RRBIs are partly caused by differential sensory processing and difficulty switching attention [53]. In addition to this, the orbitofrontal cortex is involved in reward-related learning [51, 52]. It has been hypothesized that RRBIs, and ASD in general, might be a result of alterations in the brains reward-circuitry, which comprises not only the OFC, but also striatal regions [54–56]. These networks correspond to the cortico-striatal networks that have also been shown in association with repetitive behaviors in conditions other than ASD [12].

The sex-specific findings could be an indication of etiological differences underlying symptom domains of ASD in males and females. Previously, interactions between sex and ASD diagnosis were observed for white matter connectivity density of the medial parietal lobe, of which the intraparietal sulcus is a part [19]. However, in that study, sex-specific effects were not found for gray matter. The sex-specific effects in our study became more evident when analyzing the subgroup that was either concordant or discordant for ASD. Here, increased thickness in orbitofrontal, superior frontal, and parietal area was again reported mostly in females, while males exhibited only increased pallidum volume at a more lenient statistical threshold. Of note, the observed associations between RRBI’s and brain structure in the main sample and in the ASD-subset were largely overlapping. However, the additional findings of increased pallidum volume in males, and the now significant associations in the orbital gyri and superior frontal gyrus in females suggest that the ASD-pairs had the biggest impact on the associations in the main analyses, and these might have been obscured by variation in the non-ASD pairs in the main sample. The findings suggest that RRBIs might be associated with different brain networks in females and males, with fronto-parietal networks being altered in females, while fronto-striatal networks are altered in males. This finding corresponds to that of Supekar, who also report mostly cortical regions to correlate with RRBIs in girls, while the putamen correlated with RRBIs in boys [20]. At the same time, reduced volume of the inferior frontal gyrus in relation to repetitive symptoms has been found also in males with ASD [13]. Further, although structural changes in the subcortical areas appear common in males with ASD [14, 20, 43], functional activation differences during motor learning tasks in parietal networks, correlating with RRBI symptoms [57] and activation during temporal delay discounting in the ventromedial PFC and subcortical regions [56] are found in males with ASD. Thus, it is possible that in males, functional activity differences associated with RRBIs are found regardless of brain structure, while in females, a change in brain structure might be necessary for a change in RRBI on the behavioral level. This would explain the lack of finding of structural changes in the male sample overall. Functional neuroimaging studies involving females with ASD should further elucidate these mechanisms.

Other than inherent etiological differences between males and females, an explanation of our sex-specific findings could be that the variance in brain structure within pairs was greater for females, therefore leading to significant associations in females but not males. Such increased brain structure differences, in combination with comparable differences in RRBI symptoms themselves, suggest greater cerebral and behavioral impairment in females for similar symptom levels. This observation might be a consequence of camouflaging. This entails that females need to have more severe RRBIs before even being noticed by their environment. Camouflaging leads to an underestimation of the true severity of autistic symptoms in females [58]. In fact, females might have different types of restricted interests that might be regarded by caregivers as less atypical [59]. Thus, the true symptom levels of the females in our sample might have been higher than scored, which could in turn be linked to stronger or different brain anatomy alterations that are found only in the most impaired females. Indeed, for the social symptom domain, females that displayed greater camouflaging had functional brain activation patterns that were more similar to that of typically developing girls [60]. Thus, we hypothesize that stronger changes in brain structure are needed to lead to a change in functional activation, and thus inability to camouflage their problems. Hence, observable RRBIs as in our study might be the result of the more severe brain alterations. Replication of our results in independent samples with assessments of explicitly high sensitivity to RRBIs in females is therefore desirable.

An additional alternative explanation might be that the observed reductions in volume in females are related to a more general and unspecific severity of autism symptomatology. However, re-running our analyses while regressing out highly correlated variance of other autism symptom domains and traits, thickness of both the intraparietal sulcus and orbital gyri were still associated with RRBI symptoms in females. Moreover, similar associations were observed when using RRBIs estimated with SRS-2. Compared to the SRS-2, assessing autistic-like traits in a short time frame (6 months), the ADI-R collects clinical RRBI symptoms and we used scores reflecting lifetime behaviors. Therefore, our patterns of RRBIs findings on the ADI-R and SRS-2 might indicate certain alterations in anatomy of the intraparietal sulcus and orbital gyri that are clinically relevant and robust for current or past presence of symptoms.

Finally, future research is required to specifically assess which genetic and environmental factors contribute to neuroanatomical alterations in females with ASD and whether females are more sensitive to non-shared environmental factors compared to males. Non-shared environmental factors could in this case also entail the repetitive behaviors themselves, which, when present at an early age, reinforce preexisting structural alterations [61]. Indeed, presence of RRBIs at preschool age has been shown to be associated with brain structure alterations in childhood and adolescence [61]. Further, directly assessing the influence of non-shared environmental factors would require a sample consisting of only monozygotic twins. Due to lack of power, we were unable to conduct meaningful analyses on the subsample of monozygotic twin pairs only, thus dizygotic and monozygotic twins were collapsed in the present study. However, the within-pair design does include implicit correction for age, sex, socio-economic background, and 100% of genes in the MZ twins and approximately 50% of genes in the DZ twins. Therefore, our results are more robust against environmental and partly genetic variation that otherwise might obscure smaller associations.

Taken together, our results point at the importance of investigating the female ASD phenotype, both on a behavioral and a neurobiological level, in order to understand the male and the female expression of the disorder. If future research is able to identify non-shared environmental factors that are differentially influencing the development of ASD in males and females, these could be targeted by interventions and give us more awareness of potential ASD risk factors for each sex.

## Limitations

Although our study benefits from a unique sample of twins and a thorough clinical assessment and MRI analyses, some issues need to be addressed that warrant caution in interpreting the results. Although the total twin sample is large, the regression coefficient of the within-pair analysis is influenced only by the 37 pairs (16 female) that differed by at least one point on RRBIs, limiting the power of the sex-specific within-pair analysis. In addition, while the variability of symptoms on the ADI-R C and SRS-2 AM were adequate, the mean values of RRBIs in our sample were quite low, thus neuroanatomical differences might become more apparent in samples where the difference in RRBs is larger and participants exhibited more severe RRBs. However, our within-pair design enhances sensitivity for small associations that are otherwise masked by between pair variability and genetic variation. Indeed, when we re-ran our analyses across pairs, that is simply investigating the association between RRBIs and brain structure in this cohort, we found fewer significant results, showing that the within-pair design enhances our sensitivity to small differences. Hence, the within-pair analysis increases the ability to detect neural correlates of RRBs which might be subtler compared to the effects of age and shared genetic and environmental factors.

In addition, the ADI-R assesses if RRBI’s have ever occurred in the participant’s lifetime, which means they do not necessarily need to be present now. However, as we replicate our results with the SRS-2 RRB scores, we believe that the ADI-R is an adequate measure of the impact of RRBI symptoms on brain structure.

Further, our sample size did not permit to assess monozygotic twin pairs separately. The ratio between MZ/DZ was not exactly the same between males (50/38) and females (42/20), with the male group consisting of relatively more DZ twins, thus limiting our assessment of the impact of non-shared environmental factors in males. However, this difference was not statistically significant. Further, ADHD was twice as common among the males compared to females. Although this difference was not significant either, it needs to be taken into account because symptoms of ADHD such as inattention are probably related to RRBs [62]. This would only be a problem if the higher incidence of ADHD in males would have led to a smaller within-pair difference in RRBs in males compared to females. However, this was not the case; the within-pair difference in RRBs was comparable between the sexes. It must be noted that, although our within-pair design compares twins of the same age, a wide age range could still have influenced the outcomes. For example, age-related brain changes might be dependent on the presence of RRBI symptoms [12] or follow a different pattern in ASD compared to controls [63]. Therefore, the within-pair brain differences related to RRBIs might depend on age. Although males and females did not differ on average age in our sample, females with an ASD diagnosis were older compared to diagnosed males (2.5 years in discordant pairs, 6 years in concordant pairs). The ASD pairs contribute most to the differences in RRBI’s and it is therefore possible that the observed differences were driven by the older female subjects with ASD. This does not limit the validity of the findings in the female group, but we cannot exclude the possibility that similar within-pair effects would be observed in older male subjects with ASD. However, testing the interaction between age and RRBIs on anatomy in a linear model resulted only in significant findings in regions that were mostly not associated with RRBs in either sex: superior precentral sulcus and superior parietal sulcus and could therefore not have confounded our findings. However, the right postcentral gyrus also showed an interaction with age, and we did find increased thickness of this gyrus in females in the ASD-subsample. As age might also affect the degree of relatedness between twins, future studies are needed to investigate sex effects on brain structure in ASD while additionally stratifying for age—requiring a larger sample than assessed in the current study.

Further, we allowed small segmentation errors in our data-set, due to the young age of the sample. Segmentation errors are known to be able to influence estimates of cortical thickness. Therefore, we might have missed significant associations due to noise. Re-running our analyses on a subset with high data quality replicated our initial findings, indicating that they were probably robust to quality issues. However, this replication generated additional regions showing increased cortical thickness in association with RRBIs in females. There were a few regions related to RRBIs in males as well. Thus, quality issues due to movement might have obscured some results in the main analyses, particularly in the males, as they were younger and younger participants moved more. Indeed, in the high-quality male subsample, we did see altered structure of the right supramarginal gyrus, left lateral orbital sulcus, and left anterior cingulate cortex. The overall pattern of results remained similar though, with increased thickness associated with RRBIs in particular in females.

Finally, our choice of ROIs is a compromise between reducing the number of comparisons and enhancing sensitivity for thus far unknown associations between RRBIs and brain structure, in particular in females. Thus, the number of ROIs chosen are relatively many since the brain correlates of RRBIs are not well established and we did not want to miss potentially meaningful association. At the same time, our choice of ROIs reduced the number of comparisons considerably compared to a whole-brain approach. Further, in order to not miss small but meaningful changes within pairs, we chose the rather lenient false discovery rate (FDR)-corrected *p* value instead of a more stringent family-wise error (FEW) correction. However, replication in a larger sample is therefore needed to confirm our findings.

## Conclusion

In conclusion, this twin study shows that quantified features RRBI are mostly associated with brain anatomy alterations in females. The results add evidence to the hypothesis that there are etiological differences underlying ASD between males and females.

## Supplementary information

**Additional file 1: Figure S1.** Within Pair distributions of ADI-R C scores. A within-pair difference per pair consists of the difference in score for RRBIs on ADI-R between 2 twins in a pair. The within-pair difference ranged from 0 to 5 points (B panel) in the cohort. **Figure S2.** The figure illustrates the within-pair difference model that was implemented in the analyses. The example shows within-pair difference associations in 3 twin pairs. Each line connects 2 individuals from one twin pair. In these examples, the individuals with a higher RRBI score on ADI-R compared to their co-twin, also had smaller right cerebellar cortex volumes. **Figure S3.** Within-pair association between RRBIs on ADI-R scores and thickness of the right intraparietal sulcus in males and females. Each dot represents one twin pair. For females (light-grey), there was a significant positive within-pair association between RRBIs on ADI-R and thickness of the right intraparietal sulcus.

**Additional file 2: Table S7A.** Within-pair associations between cortical volume, surface area and thickness of neocortical regions of interest (ROIs) and RRB symptoms from SRS-2. Estimates (B), standard deviations (SD) and q-values (q) for the within pair associations between all surface-based neocortical measures (volume, surface area and thickness) and RRBs as assessed by the SRS-2. A positive estimate indicates an increase in brain estimate related to more repetitive behavior symptoms. Bold text indicates significant associations (FDR-corrected q-value <0.05) or associations with q-value < 0.10 (FDR-corrected). **Table S7B.** Within-pair associations between cortical volume of subcortical and cerebellar regions of interest (ROIs) and RRB symptoms from SRS-2. Estimates (B), standard deviations (SD) and q-values (q) for the within pair associations between subcortical & cerebellar volumes and RRBs as assessed by the SRS-2. A positive estimate indicates an increase in brain estimate related to more repetitive behavior symptoms. Bold text indicates significant associations (*FDR-corrected q-value* <0.05) or associations with q-value < 0.10 (FDR-corrected). All results are FDR corrected. L. = Left, R. = Right. **Table S8A.** Within-pair model for RRBI’s (from ADI-R) predicting atomy of neocortical ROI’s, while controlling for social interaction deficits (from ADI-R). Estimates (B), standard deviations (SD) and q-values (q) for the linear associations between all surface-based neocortical measures (volume, surface area and thickness) and RRBs as assessed by the ADI-R, while controlling for autistic symptoms from the social domain, as assessed by the ADI-R. A positive estimate indicates an increase in brain estimate related to more repetitive behavior symptoms. Bold text indicates significant associations (*FDR-corrected q-value* <0.05) or associations with q-value < 0.10 (FDR-corrected). All results are FDR corrected. **Table S8B.** Within pair model for RRBI’s (from ADI-R) predicting atomy of subcortical and cerebellar ROI’s, while controlling for social interaction deficits (from ADI-R). Estimates (B), standard deviations (SD) and q-values (q) for the within-pair associations between subcortical measures (volumes only) and RRBs as assessed by the ADI-R, while controlling for autistic symptoms from the social domain, as assessed by the ADI-R. A positive estimate indicates an increase in brain estimate related to more repetitive behavior symptoms. Bold text indicates significant associations (*FDR-corrected q-value* <0.05) or associations with q-value < 0.10 (FDR-corrected). All results are FDR corrected. L. = Left, R. = Right. **Table S9A.** Within-pair model for RRBI’s (from SRS) predicting atomy of neocortical ROI’s, while controlling for social cognition deficits (from SRS). Estimates (B), standard deviations (SD) and q-values (q) for the within pair associations between all surface-based neocortical measures (volume, surface area and thickness) and RRBs as assessed by the SRS, while controlling for autistic symptoms from the social domain, as assessed by the SRS. A positive estimate indicates an increase in brain estimate related to more repetitive behavior symptoms. Bold text indicates significant associations (*FDR-corrected q-value* <0.05) or associations with q-value < 0.10 (FDR-corrected). All results are FDR corrected. **Table S9B.** Within-pair model for RRBI’s (from SRS) predicting atomy of subcortical and cerebellar ROI’s, while controlling for social cognition deficits (from SRS). Estimates (B), standard deviations (SD) and q-values (q) for the within pair associations between subcortical measures (volumes only) and RRBs as assessed by the SRS, while controlling for autistic symptoms from the social domain, as assessed by the SRS. A positive estimate indicates an increase in brain estimate related to more repetitive behavior symptoms. Bold text indicates significant associations (*FDR-corrected q-value* <0.05) or associations with q-value < 0.10 (FDR-corrected). All results are FDR corrected. L. = Left, R. = Right. **Table S10A.** Linear model assessing the interaction between age and RRBI symptoms (ADI-R) and neocortical regions of interest. Estimates (B), standard deviations (SD) and q-values (q) for the linear interaction associations between age (years) and RRBIs as assessed by the ADI-R on neocortical volume, surface area and thickness. Bold text indicates significant associations (*FDR-corrected q-value* <0.05) or associations with q-value < 0.10 (FDR-corrected). All results are FDR corrected. **Table S10B.** Linear model assessing the interaction between age and RRBI symptoms (ADI-R) and subcortical regions of interest. Estimates (B), standard deviations (SD) and q-values (q) for the linear interaction associations between age (years) and RRBIs as assessed by the ADI-R on subcortical volumes. Bold text indicates significant associations (*FDR-corrected q-value* <0.05) or associations with q-value < 0.10 (FDR-corrected). All results are FDR corrected. L. = Left, R. = Right.

## Data Availability

The datasets generated and/or analyzed during the current study are not publicly available. Data are part of a large ongoing collaborative project with currently privileged publication rights by collaborators. Data are available from the corresponding author on reasonable request.

## Abbreviations

ABIDE: Autism brain imaging data exchange
ACC: Anterior cingulate cortex
ADI-R: Autism Diagnostic Interview-Revised
ASD: Autism spectrum disorder
CSF: Cerebral spinal fluid
DSM: Diagnostic and statistical manual
DZ: Dizygotic
FAST: FMRIB’s Automated Segmentation Tool within FMRIB's Software Library
FDR: False discovery rate
FNIRT: FMRIB’s Nonlinear Image Registration Tool
GEE: Generalized estimating equation
GM: Grey matter
MNI: Montreal Neurological Institute
MZ: Monozygotic
RATTS: Roots of Autism and ADHD Twin Study Sweden
RDoC: Research domain criteria
RRBI: Restricted and repetitive behaviors and interests
SRS: Social Responsiveness Scale
WM: White matter
