# Supplementary material for: Sex differences in brain structure: a twin study on restricted and repetitive behaviors in twin pairs with and without autism
Source: Mol Autism. 2019 Dec 31;11:1. doi: 10.1186/s13229-019-0309-x (PMC6937723; doi:10.1186/s13229-019-0309-x)
Supplement: Supplementary file 1 — Additional file 1: Figure S1. Within Pair distributions of ADI-R C scores. A within-pair difference per pair consists of the difference in score for RRBIs on ADI-R between 2 twins in a pair. The within-pair difference ranged from 0 to 5 points (B panel) in the cohort. Figure S2. The figure illustrates the within-pair difference model that was implemented in the analyses. The example shows within-pair difference associations in 3 twin pairs. Each line connects 2 individuals from one twin pair. In these examples, the individuals with a higher RRBI score on ADI-R compared to their co-twin, also had smaller right cerebellar cortex volumes. Figure S3. Within-pair association between RRBIs on ADI-R scores and thickness of the right intraparietal sulcus in males and females. Each dot represents one twin pair. For females (light-grey), there was a significant positive within-pair association between RRBIs on ADI-R and thickness of the right intraparietal sulcus. [file 13229_2019_309_MOESM1_ESM.docx]

**SUPPLEMENTARY RESULTS**

1. Behavior

*1.1 Whole Cohort: Across subjects’ associations between RRBIs on the ADI-R, SRS-2 RBB subscale, SRS-2 total score and IQ*

While controlling for age and IQ, autistic trait severity (SRS-2 total score) predicted RRBIs on the ADI-R in both sexes (Females: B= 0.026, p<0.001; Males: B=0.037, p<0.001). Next, we aimed to investigate the reliability of our repetitive behavior measures, by testing if the two separate estimates of RRBIs were associated with each other. RRBIs on the ADI-R predicted SRS-2 RRB scores in both sexes (Females: B=2.27, p=0.00016; Males: B=2.20, p<0.001). Further, RRBIs on the ADI-R and SRS-2 correlated significantly with each other (All: Spearman's rho=0.59, p<0.001; Females Spearman's rho=0.58, p<0.001; Males Spearman's rho=0.60, p<0.001). Finally, we investigated if IQ could be a confounding factor in our analyses, by testing its association with RRBIs and sex. IQ did not predict RRBI scores from ADI-R or SRS-AM, nor was there an interaction between Sex and IQ on RRBIs. Therefore, IQ did not seem to be related to RRBIs in any of the sexes.

*1..2 Within-pair associations between RRBIs on the ADI-R, SRS-2 RBB subscale, SRS-2 total score and IQ*

Similar results were obtained in the within-pair analyses, with higher autistic trait severity (SRS-2 totals) predicting ADI-R RRBIs in both sexes (Females: B=0.031, p=0.0024; Males: B=0.030, p<0.001) and ADI-R RRBIs predicting SRS-2 RRB scores (Females: B=0.12, p=0.0066; Males: B=0.14, p<0.001), with no significant effect of IQ on either RRBIs on the ADI-R or SRS-2. When assessing a subset consisting of pairs of which at least one twin had a clinically relevant RRBI score (ADI-R RRBI =>2), IQ did not predict RRBI scores on either of the two scales in either of the sexes.

2. Neuroanatomy

*Interactions between RRBIs (ADI-R) and age on neuroanatomy of motor networks*

There were significant age x RRBI interaction-effects on volume of the right superior precentral sulcus, right postcentral gyrus and thickness of the superior parietal sulcus, in addition to an association at q<0.1 for left amygdala volume. See Supplementary Table 10A & B.

2.2 Post-hoc analyses: Associations between RRBIs and brain anatomy, corrected for other autistic symptoms domains (SRS-2 social cognition and ADI-R reciprocal social interaction)

We aimed to assess if the observed alterations in brain anatomy were specific to the domain of RRBIs, and not explained by other autism symptoms that were correlated with RRBIs on the ADI-R and SRS-2. Therefore, we assessed the associations between RRBIs on both scales and brain anatomy, while controlling for the SRS-2 social cognition subscale and ADI-R reciprocal social interaction domain scores.

*2.2.1 Associations between RRBIs and social cognition on the SRS-2, and RRBIs and reciprocal social interaction on the ADI-R*

First, we assessed the behavioral associations between the RRBI scales and their respective social domain sub-scales. Across all female subjects, SRS-2 RRBs correlated significantly with SRS-2 social cognition scores (Spearman's rho=0.85, p<0.001), and ADI-R RRBIs correlated significantly with ADI-R reciprocal social interaction scores (Spearman's rho=0.53, p<0.001). Across all male subjects, SRS-2 RRBs correlated significantly with SRS-2 social cognition scores (Spearman's rho=0.77, p<0.001) and ADI-R RRBIs correlated significantly with ADI-R reciprocal social interaction scores (Spearman's rho=0.61, p<0.001).

*2.2.2 Within pairs: ADI-R RRBIs predicting brain anatomy, with the additional covariate ADI-R reciprocal social interaction*

In females, ADI-R RRBIs, while controlling for ADI-R reciprocal social interaction scores, were associated with increased volume of the right orbital gyrus (B=161.09, q=0.007) and right postcentral gyrus (B=154.48, q=0.003), and with increased thickness of the right postcentral sulcus (B=0.026, q=0.031) and right intraparietal sulcus (B=0.041, q=0.008). There were associations at q<0.1 for increased thickness of the right inferior frontal orbital gyrus (B=0.075, q=0.055) and reduced surface area of the right intraparietal sulcus (B=-115.97, q=0.075). Further, in males, RRBI symptoms were associated with decreased volume of the right cerebellum cortex (B=-1092.29, q=0.014) and, at a threshold of q<0.1, for decreased volume of the left cerebellum cortex (B=-1198.49, q=0.070), and increased volume of the right inferior frontal gyrus (B=81.99, q=0.093). See Supplementary Table 8A and B.

*2.2.3 Within Pairs: SRS-2 RRBs predicting brain anatomy with the additional covariate SRS-2 social cognition*

Further, when controlling for current social cognition impairments (SRS-SKOG), to test if the observed associations between brain structure and current RRBI’s were specific to that symptom domain, in females there was an association for reduced surface area of the left lateral orbital sulcus (B=-8.13, q=0.003) and increased thickness of the left angular gyrus (B=0.023, q=0.018), in addition to associations at a threshold of q<0.1 for reduced thickness of the bilateral middle frontal sulcus (Left: -0.009, q=0.071; Right: B=-0.011, q=0.067), right postcentral gyrus (B=-0.017, q=0.056), right superior frontal gyrus (B=-0.009, q=0.055), as well as increased surface area of the right supramarginal gyrus (B=33.04, q=0.063), right superior parietal gyrus (B=26.44, q=0.066), but reduced surface area of the bilateral angular gyri (Left: B=-33.04, q=0.054; Right: B=-31.05, q=0.051). In males, there were significant associations between RRBs and reduced surface area of the right precentral gyrus (B=-27.92, p=0.002) and right central sulcus (B=-32.08, p=0.032). See Supplementary Table 9A and B.

2.3 Associations between RRBIs (ADI-R C) and brain structure in specific sub-sets of participants

*2.3.1 Within-Pairs: ADI-R C RRBIs predicting brain anatomy in subjects discordant or concordant for ASD*

To test if the associations between brain structure and RRBIs on ADI-R C were mostly driven by subjects with an ASD diagnoses, we re-ran the analyses on a subset of participants that were either concordant (n= 12 subjects, 6 males) or discordant (n=40 subjects, 28 males) for ASD. In females, RRBIs were associated with increased thickness of the left lateral orbital sulcus (B=0.110, q=0.002), right orbital gyrus (B=0.070, q=0.016), left superior frontal gyrus (B=0.043, q=0.003) and right intraparietal sulcus (B=0.032, q=0.013). In addition, there were associations at a threshold of q<0.1 for females between RRBIs and increased volume of the right orbital gyrus (B=166.73, q=0.059), increased thickness of the right postcentral gyrus (B=0.039, q=0.087) and reduced volume of the left postcentral sulcus (B=-208.12, q=0.059). In males, there increased volume of the bilateral pallidum (Left: 23.19, q=0.096; Right: B=31.51, q=0.096) in association with RRBIs was observed at a threshold of q<0.1.

*2.3.2 Within-Pairs: ADI-R C RRBIs predicting brain anatomy in subjects with very high data quality*

In the main analyses we had included participants who had received both quality scores 1 and 2. A score of 2 entailed that some minor segmentation errors were included. In order to investigate the impact of these errors on our results, we re-ran the analyses on a subsample of subjects who had received a FreeSurfer quality score of 1 (n=70 subjects, 34 males). In females, RRBIs were associated with increased thickness of the left lateral orbital sulcus (B=0.094, q=0.002), left middle frontal gyrus (B=0.036, q=0.031), left inferior frontal orbital gyrus (B=0.069, q=0.022), right postcentral gyrus (B=0.038, q=0.036), sulcus (B=0.038, q<0.001), left superior frontal gyrus (B=0.059, q<0.001), and bilateral intraparietal sulci, (Left: B=0.045, q<0.001; Right: B=0.053, q=0.027) in addition to reduced surface area of the left superior frontal gyrus (B=-192.15, q=0.010). Further, at a threshold of q0.1, there was an association between RRBIs and increased volume of the left inferior frontal orbital gyrus (B=88.10, q=0.059) and right postcentral gyrus (B=235.05, q=0.053) in females.

In males, RRBIs were associated with reduced surface area of the left lateral orbital sulcus (B=-28.44, q=0.001), increased thickness of the left ACC (B=0.093, q<0.001) and increased surface area (B=130.6, q=0.035) and volume (B=842.22, q=0.018) of the right supramarginal gyrus.

2.4 Linear Associations between RRBIs (ADI-R C) and brain structure across participants

We also tested the association between RRBIs and brain structure across all pairs, i.e. without taking twinness into account. For this we used a regular linear model within the gee framework, but adjusted the standard errors to consider the non-dependence of the data points from each twin pair.

*2.4.1 Linear Associations between RRBIs and brain structure across all individuals*

Across all individuals RRBIs were not associated with brain structure.

*2.4.2 Linear Associations between RRBIs and brain structure in males and females separately*

Across all females we found that RRBIs were associated with reduced surface area of the right intraparietal sulcus (B=-93.61, q<0.001). In addition, at a threshold of q<0.1, we observed increased thickness of the left ACC in females (B=0.025, q=0.072). In males, RRBIs were not associated with brain structure.

SUPPLEMENTARY FIGURES **
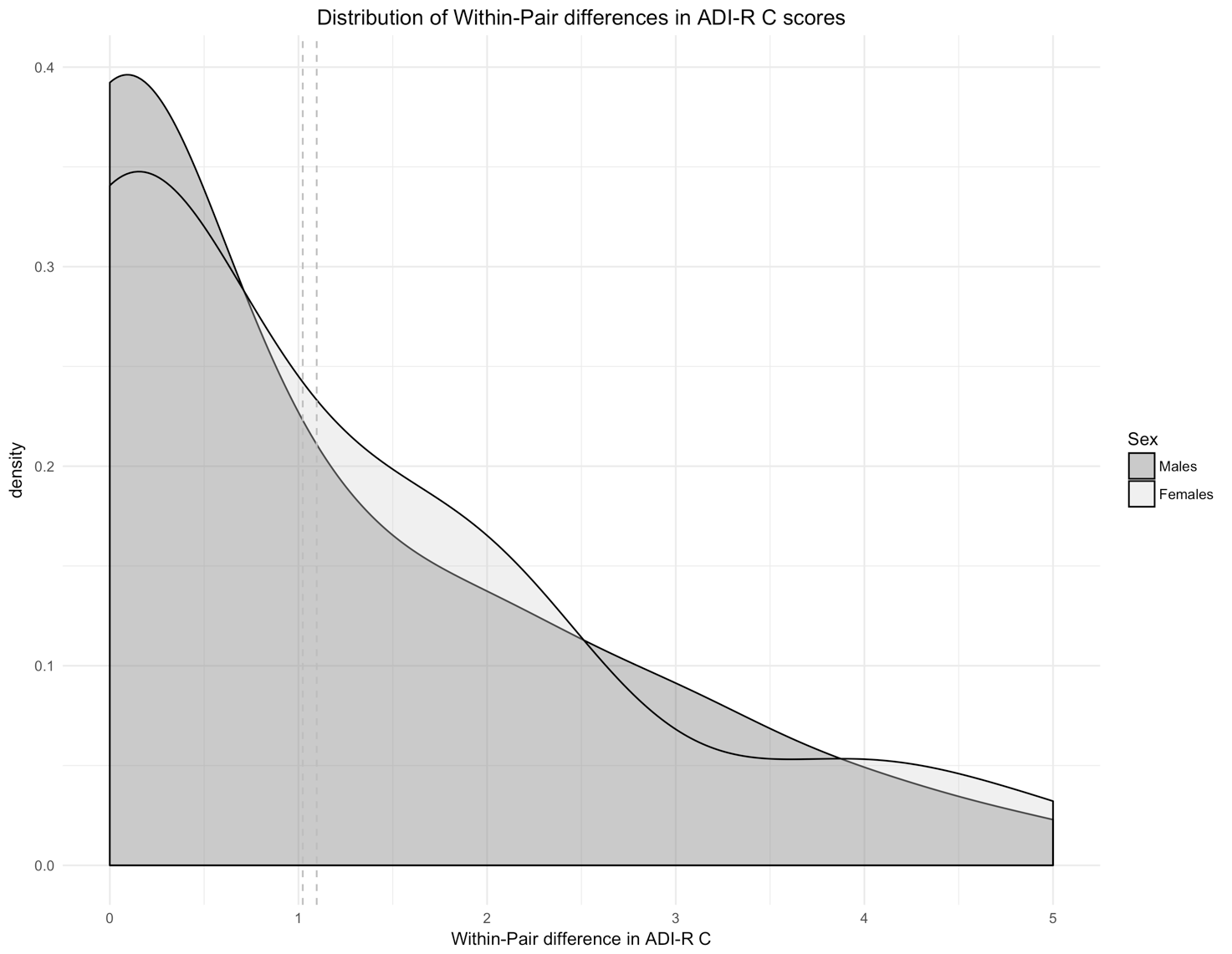

Supplementary Figure 1.** Within Pair distributions of ADI-R C scores. A within-pair difference per pair consists of the difference in score for RRBIs on ADI-R between 2 twins in a pair. The within-pair difference ranged from 0 to 5 points (B panel) in the cohort.


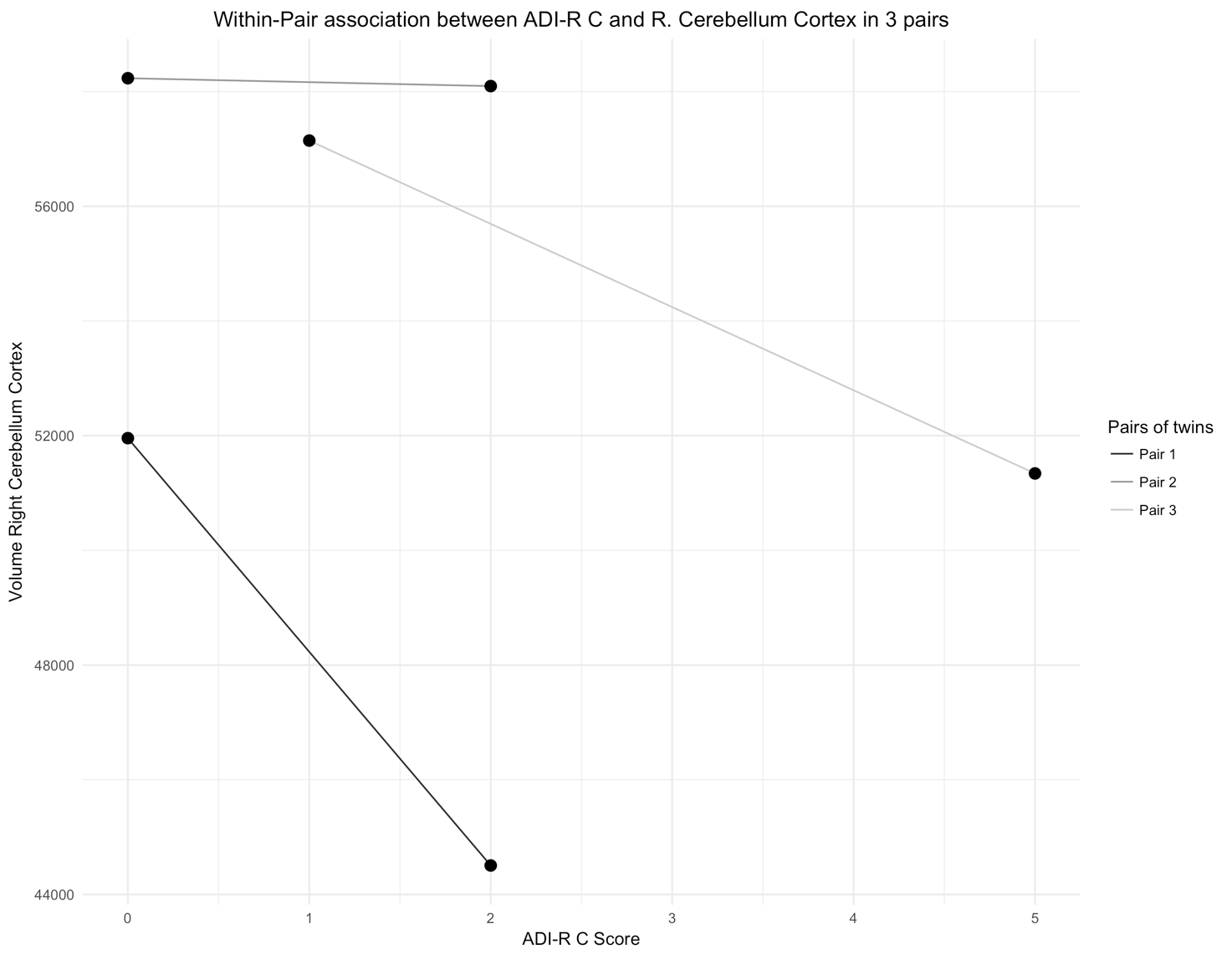


**Supplementary Figure 2.** The figure illustrates the within-pair difference model that was implemented in the analyses. The example shows within-pair difference associations in 3 twin pairs. Each line connects 2 individuals from one twin pair. In these examples, the individuals with a higher RRBI score on ADI-R compared to their co-twin, also had smaller right cerebellar cortex volumes.


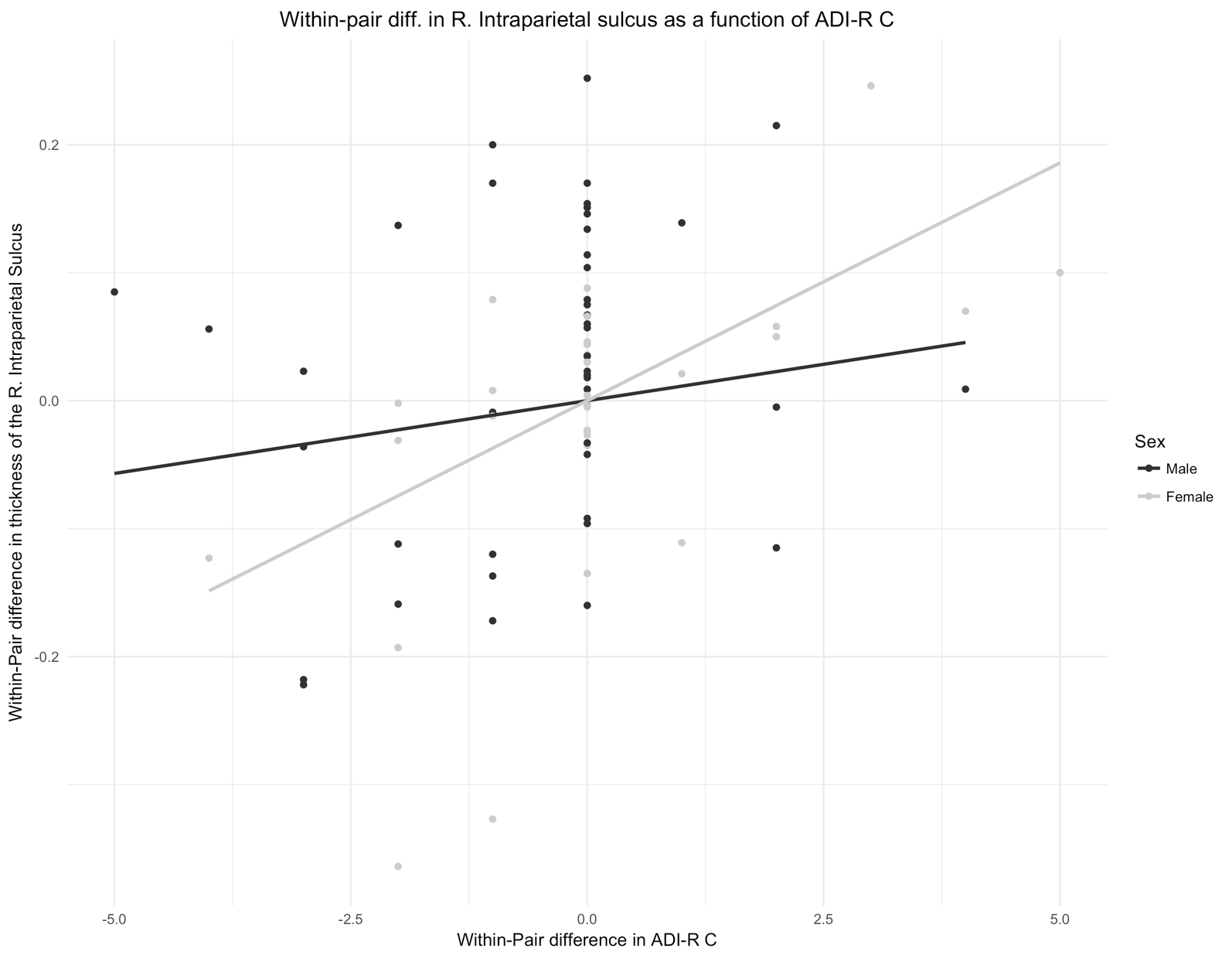


**Supplementary Figure 3.** Within-pair association between RRBIs on ADI-R scores and thickness of the right intraparietal sulcus in males and females. Each dot represents one twin pair. For females (light-grey), there was a significant positive within-pair association between RRBIs on ADI-R and thickness of the right intraparietal sulcus.

SUPPLEMENTARY TABLES

All supplementary tables can be found in a separate document called the *supplementary tables*. In each of these tables, a positive estimate indicates an increase in brain estimate related to more repetitive behavior symptoms. Bold text indicates significant associations (FDR-corrected q-value <0.05) or associations with q-value < 0.10 (FDR-corrected). All results are FDR corrected. L. = Left, R. = Right.
