# Supplementary material for: Sex differences in brain structure: a twin study on restricted and repetitive behaviors in twin pairs with and without autism
Source: Mol Autism. 2019 Dec 31;11:1. doi: 10.1186/s13229-019-0309-x (PMC6937723; doi:10.1186/s13229-019-0309-x)
Supplement: Supplementary file 2 — Additional file 2: Table S7A. Within-pair associations between cortical volume, surface area and thickness of neocortical regions of interest (ROIs) and RRB symptoms from SRS-2. Estimates (B), standard deviations (SD) and q-values (q) for the within pair associations between all surface-based neocortical measures (volume, surface area and thickness) and RRBs as assessed by the SRS-2. A positive estimate indicates an increase in brain estimate related to more repetitive behavior symptoms. Bold text indicates significant associations (FDR-corrected q-value <0.05) or associations with q-value < 0.10 (FDR-corrected). Table S7B. Within-pair associations between cortical volume of subcortical and cerebellar regions of interest (ROIs) and RRB symptoms from SRS-2. Estimates (B), standard deviations (SD) and q-values (q) for the within pair associations between subcortical & cerebellar volumes and RRBs as assessed by the SRS-2. A positive estimate indicates an increase in brain estimate related to more repetitive behavior symptoms. Bold text indicates significant associations (FDR-corrected q-value <0.05) or associations with q-value < 0.10 (FDR-corrected). All results are FDR corrected. L. = Left, R. = Right. Table S8A. Within-pair model for RRBI’s (from ADI-R) predicting atomy of neocortical ROI’s, while controlling for social interaction deficits (from ADI-R). Estimates (B), standard deviations (SD) and q-values (q) for the linear associations between all surface-based neocortical measures (volume, surface area and thickness) and RRBs as assessed by the ADI-R, while controlling for autistic symptoms from the social domain, as assessed by the ADI-R. A positive estimate indicates an increase in brain estimate related to more repetitive behavior symptoms. Bold text indicates significant associations (FDR-corrected q-value <0.05) or associations with q-value < 0.10 (FDR-corrected). All results are FDR corrected. Table S8B. Within pair model for RRBI’s (from ADI-R) predicting [file 13229_2019_309_MOESM2_ESM.docx]

**Table S7A**. Within-pair associations between cortical volume, surface area and thickness of neocortical regions of interest (ROIs) and RRB symptoms from SRS-2.

| **RRB’s from SRS – Atomy**  **“Neocortical”** | **Cortical Volume** | | **Surface Area** | | **Cortical thickness** | |
| --- | --- | --- | --- | --- | --- | --- |
|  | Males β (SD) *q* | Females β (SD) *q* | Males β (SD) *q* | Females β (SD) *q* | Males β (SD) *q* | Females β (SD) *q* |
| *Right Lateral Orbital Sulcus* | -5.85 | 2.67 | -0.38 | -0.38 | -0.004 | 0.008 |
|  | (2.66) | (4.17) | (1.25) | (2.07) | (0.003) | (0.005) |
|  | *0.253* | *0.809* | *0.962* | *0.959* | *0.831* | *0.366* |
| *Left Lateral Orbital Sulcus* | -2.69 | 2.32 | -1.26 | -3.23 | -0.004 | **0.017** |
|  | (5.1) |  | (1.77) | (1.49) | (0.006) | **(0.005)** |
|  | *0.861* | *0.809* | *0.853* | *0.328* | *0.955* | ***0.007*** |
| *Right Middle Frontal Sulcus* | -1.2 | 2.37 | 0.24 | 6.82 | -0.001 | -0.003 |
|  | (25.61) | (26.37) | (8.13) | (7.23) | (0.003) | (0.003) |
|  | *0.963* | *0.957* | *0.977* | *0.732* | *0.955* | *0.684* |
| *Left Middle Frontal Sulcus* | -20.81 | 15.3 | -5.68 | 4.04 | -0.002 | -0.004 |
|  | (13.25) | (15.31) | (4.55) | (5.36) | (0.004) | (0.003) |
|  | *0.698* | *0.801* | *0.673* | *0.812* | *0.955* | *0.512* |
| *Right Orbital Gyrus* | 16.14 | 9.37 | 5.63 | -3.78 | -0.003 | **0.013** |
|  | (11.98) | (12.9) | (3.25) | (3.09) | (0.003) | **(0.004)** |
|  | *0.780* | *0.801* | *0.527* | *0.569* | *0.831* | ***0.008*** |
| *Left Orbital Gyrus* | 8.79 | -13.73 | 0.59 | -4.49 | -0.001 | 0.007 |
|  | (12.94) | (15.05) | (2.88) | (2.66) | (0.006) | (0.008) |
|  | *0.861* | *0.801* | *0.977* | *0.390* | *0.955* | *0.684* |
| *Right Middle Frontal Gyrus* | 40.1 | 63.65 | 7.46 | 15.86 | 0.0005 | -4e-05 |
|  | (41.3) | (34.2) | (9.32) | (9.57) | (0.002) | (0.003) |
|  | *0.861* | *0.801* | *0.853* | *0.390* | *0.955* | *0.992* |
| *Left Middle Frontal Gyrus* | 13.92 | 3.77 | 7.92 | -1.46 | -0.004 | 0.0006 |
|  | (24.37) | (43.25) | (5.6) | (10.08) | (0.003) | (0.003) |
|  | *0.861* | *0.957* | *0.629* | *0.959* | *0.831* | *0.905* |
| *Right Inferior Frontal Orbital Gyrus* | 8.25 | 3.29 | 1.8 | -0.93 | 0.002 | 0.011 |
|  | (5.94) | (14.59) | (1.04) | (2.43) | (0.007) | (0.005) |
|  | *0.780* | *0.929* | *0.527* | *0.921* | *0.955* | *0.160* |
| *Left Inferior Frontal Orbital Gyrus* | 8.64 | 1.67 | 1.43 | -1.02 | 0.0039 | 0.004 |
|  | (4.11) | (5.38) | (1.18) | (1.8) | (0.004) | (0.006) |
|  | *0.258* | *0.929* | *0.673* | *0.848* | *0.831* | *0.707* |
| *Right Anterior Cingulate Cortex* | 11.87 | 16.69 | 1.2 | 9.02 | 0.0007 | -0.001 |
|  | (16.82) | (21.95) | (7.62) | (7.02) | (0.003) | (0.003) |
|  | *0.861* | *0.801* | *0.977* | *0.569* | *0.955* | *0.707* |
| *Left Anterior Cingulate Cortex* | 5.17 | 18.84 | 0.32 | 4.99 | -0.001 | 0.002 |
|  | (16.86) | (12.24) | (6.07) | (3.2) | (0.003) | (0.003) |
|  | *0.898* | *0.801* | *0.977* | *0.428* | *0.955* | *0.707* |
| *Right PostCentral Gyrus* | 4.97 | -7.43 | -4.27 | 2.72 | 0.006 | -0.005 |
|  | (14.66) | (19.98) | (3.91) | (5.38) | (0.004) | (0.004) |
|  | *0.898* | *0.929* | *0.769* | *0.848* | *0.831* | *0.426* |
| *Left PostCentral Gyrus* | 7.9 | 14.78 | 7.33 | 3.32 | -0.002 | 0.003 |
|  | (10.44) | (17.42) | (5.05) | (5.77) | (0.005) | (0.004) |
|  | *0.861* | *0.801* | *0.629* | *0.848* | *0.955* | *0.707* |
| *Right PostCentral Sulcus* | **-48.93** | 13.05 | **-21.74** | 0.59 | 0.002 | 0.002 |
|  | **(15.55)** | (13.03) | **(6.91)** | (5.14) | (0.002) | (0.002) |
|  | ***0.059*** | *0.801* | ***0.060*** | *0.959* | *0.836* | *0.611* |
| *Left Post Central Sulcus* | -3.29 | -20.28 | -0.5 | -8.83 | -0.002 | 0.006 |
|  | (17.37) | (13.12) | (8.14) | (6.49) | (0.002) | (0.004) |
|  | *0.898* | *0.801* | *0.977* | *0.569* | *0.831* | *0.426* |
| *Right PreCentral Gyrus* | -11.6 | -3.11 | -4.27 | 2.81 | -0.0009 | -0.003 |
|  | (20.35) | (13.08) | (4.82) | (4.29) | (0.005) | (0.005) |
|  | *0.861* | *0.929* | *0.846* | *0.848* | *0.955* | *0.707* |
| *Left PreCentral Gyrus* | -11.99 | 16.17 | 2.19 | 5.36 | -0.004 | -0.001 |
|  | (19.9) | (16.56) | (4.08) | (5.99) | (0.004) | (0.002) |
|  | *0.861* | *0.801* | *0.886* | *0.741* | *0.831* | *0.707* |
| *Right Inferior PreCentral Sulcus* | -11.76 | 5.61 | -7.45 | 2.49 | 0.001 | -0.003 |
|  | (10.88) | (13.32) | (4.36) | (4.4) | (0.003) | (0.004) |
|  | *0.861* | *0.929* | *0.527* | *0.848* | *0.955* | *0.707* |
| *Left Inferior PreCentral Sulcus* | 25.06 | 0.06 | 4.93 | 1.31 | 0.003 | 0.0006 |
|  | (10.4) | (14.34) | (3.16) | (5.28) | (0.002) | (0.002) |
|  | *0.253* | *0.997* | *0.610* | *0.934* | *0.831* | *0.885* |
| *Right Superior PreCentral Sulcus* | 1.31 | -21.47 | 1 | -10.26 | -0.003 | -0.002 |
|  | (8.23) | (16.54) | (3.45) | (8.3) | (0.003) | (0.005) |
|  | *0.898* | *0.801* | *0.962* | *0.569* | *0.929* | *0.707* |
| *Left Superior PreCentral Sulcus* | 2.18 | 3.23 | 1.74 | 0.07 | -0.002 | -0.0002 |
|  | (12.32) | (11.31) | (4.99) | (4.76) | (0.003) | (0.004) |
|  | *0.898* | *0.929* | *0.962* | *0.988* | *0.955* | *0.979* |
| *Right Central Sulcus* | 6.84 | 4.85 | 0.71 | 1.02 | 0.002 | -0.0005 |
|  | (12.66) | (6.63) | (5.81) | (3.11) | (0.002) | (0.002) |
|  | *0.861* | *0.801* | *0.977* | *0.921* | *0.831* | *0.867* |
| *Left Central Sulcus* | -4.56 | 11.24 | -3.92 | 2.29 | 0.0003 | 0.0009 |
|  | (10.55) | (11.97) | (4.2) | (6.69) | (0.002) | (0.002) |
|  | *0.898* | *0.801* | *0.846* | *0.921* | *0.955* | *0.707* |
| *Right Superior Frontal Sulcus* | 24.7 | 24.67 | 4.06 | 2.53 | 0.005 | 0.005 |
|  | (20.9) | (27.28) | (7.78) | (9.56) | (0.002) | (0.004) |
|  | *0.861* | *0.801* | *0.886* | *0.934* | *0.295* | *0.476* |
| *Left Superior Frontal Sulcus* | 4.95 | 13.39 | 1.93 | 6.41 | -0.003 | -0.005 |
|  | (19.93) | (22.28) | (6.76) | (6.44) | (0.002) | (0.002) |
|  | *0.898* | *0.809* | *0.962* | *0.719* | *0.831* | *0.221* |
| *Right Superior Frontal Gyrus* | -6.71 | 46.96 | -1.72 | 14.97 | 0.0001 | -0.003 |
|  | (37.11) | (32.34) | (9.1) | (7.8) | (0.002) | (0.002) |
|  | *0.898* | *0.801* | *0.977* | *0.332* | *0.955* | *0.426* |
| *Left Superior Frontal Gyrus* | -69.53 | -37.02 | -21.27 | -16.89 | 0.003 | 0.005 |
|  | (30.95) | (39.42) | (8.84) | (9.06) | (0.002) | (0.002) |
|  | *0.253* | *0.801* | *0.290* | *0.332* | *0.831* | *0.120* |
| *Right Supramarginal Gyrus* | -10.87 | 34.44 | -14.54 | **18.69** | 0.009 | -0.0039 |
|  | (29.91) | (16.43) | (6.86) | **(5.01)** | (0.005) | (0.006) |
|  | *0.898* | *0.801* | *0.407* | ***0.007*** | *0.831* | *0.707* |
| *Left Supramarginal Gyrus* | -11.27 | -50.03 | -5.84 | -17.12 | 0.003 | 0.007 |
|  | (20.65) | (30.65) | (8.01) | (9.27) | (0.004) | (0.006) |
|  | *0.861* | *0.801* | *0.853* | *0.332* | *0.836* | *0.570* |
| *Right Superior Parietal Gyrus* | -12.29 | 6.24 | -2.81 | 6.48 | 0.0003 | -0.002 |
|  | (18.17) | (33.43) | (5.59) | (8.15) | (0.004) | (0.003) |
|  | *0.861* | *0.929* | *0.886* | *0.807* | *0.955* | *0.707* |
| *Left Superior Parietal Gyrus* | -3.74 | 22.36 | 4.74 | 0.72 | -7e-04 | 0.006 |
|  | (15.32) | (29.84) | (6.98) | (8.46) | (0.005) | (0.005) |
|  | *0.898* | *0.801* | *0.853* | *0.959* | *0.955* | *0.570* |
| *Right Intraparietal Sulcus* | 18.36 | -25.81 | 11.57 | -15.6 | 0.002 | 0.006 |
|  | (16.74) | (25.82) | (8.58) | (13.19) | (0.002) | (0.003) |
|  | *0.861* | *0.801* | *0.639* | *0.569* | *0.831* | *0.252* |
| *Left Intraparietal Sulcus* | -15.04 | 4.45 | -6.31 | -4.69 | -0.0009 | **0.006** |
|  | (16.74) | (20.43) | (8.15) | (8.97) | (0.002) | **(0.002)** |
|  | *0.861* | *0.929* | *0.853* | *0.848* | *0.955* | ***0.049*** |
| *Right Angular Gyrus* | 27.9 | -25.11 | 7.2 | -16.46 | 0.0008 | 0.008 |
|  | (39.6) | (30.25) | (7.83) | (7.87) | (0.005) | (0.006) |
|  | *0.861* | *0.801* | *0.846* | *0.328* | *0.955* | *0.476* |
| *Left Angular Gyrus* | -24.21 | -11.36 | -4.43 | -14.17 | -0.0005 | 0.008 |
|  | (33.1) | (17.91) | (8.46) | (5.12) | (0.005) | (0.005) |
|  | *0.861* | *0.809* | *0.886* | *0.102* | *0.955* | *0.417* |

Estimates (B), standard deviations (SD) and q-values (q) for the within pair associations between all surface-based neocortical measures (volume, surface area and thickness) and RRBs as assessed by the SRS-2. A positive estimate indicates an increase in brain estimate related to more repetitive behavior symptoms. Bold text indicates significant associations (FDR-corrected q-value <0.05) or associations with q-value < 0.10 (FDR-corrected).

**Table S7B**. Within-pair associations between cortical volume of subcortical and cerebellar regions of interest (ROIs) and RRB symptoms from SRS-2.

| **RRB’s from SRS – Atomy**  **“Subcortical”** | **SubCortical Volume** | | | | |
| --- | --- | --- | --- | --- | --- |
| ROI's Subcortical | Males B (SD) q | Females B (SD) q | ROI's Subcortical | Males B (SD) q | Females B (SD) q |
| *L. Thalamus Proper* | 10.98 | -5.73 | *L. Cerebellum White* | 21.73 | 30.39 |
|  | (12.9) | (9.69) |  | (21.93) | (40.68) |
|  | *0.801* | *0.817* |  | *0.734* | *0.681* |
| *L. Caudate* | 11.83 | -10.95 | *L. Cerebellum Cortex* | -60.31 | -69.21 |
|  | (7.27) | (8.59) |  | (97.67) | (56.15) |
|  | *0.519* | *0.787* |  | *0.734* | *0.653* |
| *L. Putamen* | -6.73 | -2.7 | *R. Cerebellum White* | 15.43 | 10 |
|  | (8.21) | (7.16) |  | (17.55) | (28.18) |
|  | *0.801* | *0.817* |  | *0.734* | *0.723* |
| *L. Pallidum* | -0.66 | 4.77 | *R. Cerebellum Cortex* | -44.77 | -36.43 |
|  | (2.3) | (5.71) |  | (88.22) | (60.52) |
|  | *0.905* | *0.817* |  | *0.734* | *0.681* |
| *L. Amygdala* | -3.01 | 1.57 | *Somato Motor* | 1.45 | 1.94 |
|  | (3.14) | (3.5) |  | (1.52) | (3.39) |
|  | *0.801* | *0.817* |  | *0.734* | *0.681* |
| *R. Thalamus Proper* | 2.07 | 6.12 | *Somato Motor White* | 0.62 | 6.45 |
|  | (10.04) | (7.87) |  | (2.89) | (4.11) |
|  | *0.905* | *0.817* |  | *0.830* | *0.653* |
| *R. Caudate* | 1.16 | -11.55 |  |  |  |
|  | (8.25) | (8.52) |  |  |  |
|  | *0.905* | *0.787* |  |  |  |
| *R. Putamen* | -1.16 | -1.23 |  |  |  |
|  | (9.76) | (5.33) |  |  |  |
|  | *0.905* | *0.817* |  |  |  |
| *R. Pallidum* | **5.99** | -1.17 |  |  |  |
|  | **(1.71)** | (3.86) |  |  |  |
|  | ***0.005*** | *0.817* |  |  |  |
| *R. Amygdala* | 2.15 | 6.25 |  |  |  |
|  | (3.05) | (5.27) |  |  |  |
|  | *0.801* | *0.787* |  |  |  |

Estimates (B), standard deviations (SD) and q-values (q) for the within pair associations between subcortical & cerebellar volumes and RRBs as assessed by the SRS-2. A positive estimate indicates an increase in brain estimate related to more repetitive behavior symptoms. Bold text indicates significant associations (*FDR-corrected q-value* <0.05) or associations with q-value < 0.10 (FDR-corrected). All results are FDR corrected. L. = Left, R. = Right.

**Table S8A.** Within-pair model for RRBI’s (from ADI-R) predicting atomy of neocortical ROI’s, while controlling for social interaction deficits (from ADI-R).

| **RRBIs with Social Interaction Control (ADI-R)**  **“Neocortical”** | **Cortical Volume** | | **Surface Area** | | **Cortical thickness** | |
| --- | --- | --- | --- | --- | --- | --- |
|  | Males β (SD) *q* | Females β (SD) *q* | Males β (SD) *q* | Females β (SD) *q* | Males β (SD) *q* | Females β (SD) *q* |
| *Right Lateral Orbital Sulcus* | -25.22 | 31.38 | -2.75 | 3.10 | -0.010 | 0.030 |
|  | (24.26) | (19.5) | (11.24) | (10.65) | (0.023) | (0.020) |
|  | *0.860* | *0.322* | *0.975* | *0.981* | *0.829* | *0.372* |
| *Left Lateral Orbital Sulcus* | 15.87 | 53.02 | 3.96 | 5 | 0.009 | 0.060 |
|  | (19.56) | (26.72) | (9.26) | (9.51) | (0.030) | (0.041) |
|  | *0.860* | *0.228* | *0.975* | *0.899* | *0.846* | *0.395* |
| *Right Middle Frontal Sulcus* | 13.13 | -20.63 | -14.3 | -3.24 | 0.018 | 8e-04 |
|  | (99.98) | (104.16) | (28.89) | (34.04) | (0.018) | (0.019) |
|  | *0.969* | *0.950* | *0.975* | *0.981* | *0.706* | *0.966* |
| *Left Middle Frontal Sulcus* | -87.52 | -11.38 | -9.57 | -9.29 | -0.009 | -0.007 |
|  | (98.82) | (62.15) | (31.01) | (16.07) | (0.021) | (0.0180) |
|  | *0.860* | *0.950* | *0.975* | *0.899* | *0.829* | *0.802* |
| *Right Orbital Gyrus* | 75.6 | **161.09** | 8.06 | 17.7 | 0.0008 | 0.044 |
|  | (83.81) | **(45.56)** | (20.52) | (18.29) | (0.017) | (0.018) |
|  | *0.860* | ***0.007*** | *0.975* | *0.775* | *0.960* | *0.144* |
| *Left Orbital Gyrus* | **214.37** | -6.92 | 16.34 | -5.47 | 0.032 | 0.007 |
|  | **(76.69)** | (71.82) | (16.57) | (15.92) | (0.029) | (0.024) |
|  | ***0.093*** | *0.950* | *0.975* | *0.981* | *0.706* | *0.827* |
| *Right Middle Frontal Gyrus* | 72.52 | 225.01 | 1.45 | 77.21 | 0.014 | -0.016 |
|  | (180.07) | (143.24) | (45.31) | (35.47) | (0.011) | (0.018) |
|  | *0.960* | *0.322* | *0.975* | *0.213* | *0.706* | *0.501* |
| *Left Middle Frontal Gyrus* | 82.45 | 101.4 | 55.99 | 18.58 | -0.019 | -0.003 |
|  | (209.1) | (157.38) | (43.91) | (33.55) | (0.017) | (0.022) |
|  | *0.960* | *0.779* | *0.975* | *0.899* | *0.706* | *0.901* |
| *Right Inferior Frontal Orbital Gyrus* | **81.99** | -5.21 | 12.08 | -6.03 | 0.028 | **0.075** |
|  | **(28.4)** | (36.9) | (5.78) | (8.02) | (0.029) | **(0.027)** |
|  | ***0.093*** | *0.950* | *0.599* | *0.899* | *0.706* | ***0.055*** |
| *Left Inferior Frontal Orbital Gyrus* | 17.91 | 42.18 | 3.97 | 1.8 | 0.013 | 0.037 |
|  | (26.98) | (27.9) | (5.58) | (6.03) | (0.025) | (0.022) |
|  | *0.860* | *0.336* | *0.975* | *0.981* | *0.813* | *0.349* |
| *Right Anterior Cingulate Cortex* | 38.82 | -107.09 | -12.73 | -31.2 | 0.021 | 0.006 |
|  | (57.88) | (88.75) | (27.5) | (24.6) | (0.017) | (0.014) |
|  | *0.860* | *0.512* | *0.975* | *0.605* | *0.706* | *0.801* |
| *Left Anterior Cingulate Cortex* | 67.58 | 30.3 | 26.17 | -2.92 | 0.005 | 0.024 |
|  | (102.12) | (61.47) | (32.72) | (15.59) | (0.016) | (0.014) |
|  | *0.860* | *0.861* | *0.975* | *0.981* | *0.846* | *0.349* |
| *Right PostCentral Gyrus* | 106.91 | **154.48** | 1.6 | 32.56 | 0.023 | 0.021 |
|  | (99.45) | **(39.46)** | (27.88) | (18.07) | (0.022) | (0.018) |
|  | *0.860* | ***0.003*** | *0.975* | *0.369* | *0.706* | *0.412* |
| *Left PostCentral Gyrus* | 40.82 | -19.9 | 19.1 | -6.35 | 0.011 | 0.011 |
|  | (78.57) | (101.3) | (35.1) | (37.54) | (0.022) | (0.017) |
|  | *0.905* | *0.950* | *0.975* | *0.981* | *0.827* | *0.653* |
| *Right PostCentral Sulcus* | -193.10 | 77.21 | -107.08 | 15.66 | 0.009 | **0.026** |
|  | (109.8) | (76.86) | (40.94) | (33.00) | (0.014) | **(0.008)** |
|  | *0.507* | *0.597* | *0.321* | *0.915* | *0.809* | ***0.031*** |
| *Left Post Central Sulcus* | -31.53 | -62.78 | -20.2 | -21.14 | 0.006 | 0.012 |
|  | (100.00) | (51.16) | (50.96) | (23.87) | (0.017) | (0.009) |
|  | *0.968* | *0.512* | *0.975* | *0.796* | *0.846* | *0.399* |
| *Right PreCentral Gyrus* | -204.87 | -2.84 | -27.37 | 22.21 | -0.012 | -0.019 |
|  | (93.31) | (60.67) | (30.28) | (19.45) | (0.020) | (0.016) |
|  | *0.338* | *0.963* | *0.975* | *0.652* | *0.809* | *0.412* |
| *Left PreCentral Gyrus* | -101.9 | 63.4 | 1.51 | 33.1 | -0.016 | -0.020 |
|  | (138.14) | (77.6) | (25.24) | (21.77) | (0.022) | (0.009) |
|  | *0.860* | *0.666* | *0.975* | *0.463* | *0.809* | *0.186* |
| *Right Inferior PreCentral Sulcus* | -71.46 | -11.65 | -49.09 | -0.81 | 0.006 | -0.006 |
|  | (66.72) | (51.71) | (25.04) | (19.37) | (0.021) | (0.016) |
|  | *0.860* | *0.950* | *0.599* | *0.981* | *0.846* | *0.802* |
| *Left Inferior PreCentral Sulcus* | 88.68 | 21.73 | 18.03 | 1.18 | 0.008 | 0.010 |
|  | (54.96) | (61.05) | (15.42) | (23.74) | (0.013) | (0.010) |
|  | *0.548* | *0.950* | *0.975* | *0.981* | *0.809* | *0.440* |
| *Right Superior PreCentral Sulcus* | 16.42 | 10.11 | 1.97 | 15.78 | 0.002 | -0.019 |
|  | (46.6) | (56.66) | (20.48) | (25.51) | (0.012) | (0.012) |
|  | *0.966* | *0.950* | *0.975* | *0.899* | *0.902* | *0.372* |
| *Left Superior PreCentral Sulcus* | -3.65 | 81.55 | 9.32 | 23.58 | 0.007 | 0.013 |
|  | (57.78) | (41.72) | (27.26) | (17.00) | (0.012) | (0.012) |
|  | *0.973* | *0.228* | *0.975* | *0.542* | *0.809* | *0.412* |
| *Right Central Sulcus* | -3.29 | 83.37 | -5.39 | 43.84 | -0.011 | -0.010 |
|  | (97.89) | (43.84) | (42.44) | (17.2) | (0.009) | (0.008) |
|  | *0.973* | *0.229* | *0.975* | *0.130* | *0.706* | *0.398* |
| *Left Central Sulcus* | -23.74 | 37.28 | -20.19 | 18.64 | 0.0008 | -0.006 |
|  | (39.64) | (68.8) | (19.98) | (34.35) | (0.009) | (0.008) |
|  | *0.860* | *0.847* | *0.975* | *0.899* | *0.958* | *0.567* |
| *Right Superior Frontal Sulcus* | 41.47 | 319.67 | 9.78 | 97.15 | 0.018 | 0.017 |
|  | (161.17) | (128.86) | (53.06) | (36.99) | (0.013) | (0.013) |
|  | *0.969* | *0.157* | *0.975* | *0.130* | *0.706* | *0.398* |
| *Left Superior Frontal Sulcus* | 14.83 | 115.75 | -8.29 | 29.11 | 0.013 | 0.016 |
|  | (125.12) | (100.81) | (44.67) | (30.78) | (0.012) | (0.010) |
|  | *0.969* | *0.531* | *0.975* | *0.775* | *0.706* | *0.372* |
| *Right Superior Frontal Gyrus* | 340.8 | 244.74 | 69.54 | 65.7 | 0.015 | 0.010 |
|  | (185.81) | (113.93) | (46.92) | (32.53) | (0.012) | (0.010) |
|  | *0.507* | *0.205* | *0.975* | *0.261* | *0.706* | *0.432* |
| *Left Superior Frontal Gyrus* | -24.93 | -315.15 | -24.33 | -117.39 | 0.011 | 0.028 |
|  | (168.2) | (199.79) | (48.72) | (49.61) | (0.008) | (0.014) |
|  | *0.969* | *0.322* | *0.975* | *0.162* | *0.706* | *0.186* |
| *Right Supramarginal Gyrus* | 106.12 | 65.78 | -23.37 | 6.45 | 0.034 | 0.029 |
|  | (131.03) | (78.42) | (35.76) | (36.62) | (0.035) | (0.021) |
|  | *0.860* | *0.666* | *0.975* | *0.981* | *0.706* | *0.395* |
| *Left Supramarginal Gyrus* | -84.35 | -242.8 | -15.93 | -52.34 | -0.013 | -0.018 |
|  | (137.45) | (112.31) | (56.52) | (32.51) | (0.020) | (0.021) |
|  | *0.860* | *0.205* | *0.975* | *0.430* | *0.809* | *0.501* |
| *Right Superior Parietal Gyrus* | -95.51 | -105.55 | -1.88 | -40.97 | -0.017 | 0.016 |
|  | (127.73) | (111.75) | (42.94) | (33.29) | (0.018) | (0.013) |
|  | *0.860* | *0.621* | *0.975* | *0.605* | *0.706* | *0.399* |
| *Left Superior Parietal Gyrus* | -127.69 | 21.56 | 25.37 | -6.5 | -0.033 | -0.005 |
|  | (113.19) | (208.5) | (41.05) | (52.55) | (0.027) | (0.022) |
|  | *0.860* | *0.950* | *0.975* | *0.981* | *0.706* | *0.871* |
| *Right Intraparietal Sulcus* | 16.8 | -146.16 | 14.16 | **-115.97** | 0.009 | **0.041** |
|  | (149.19) | (84.36) | (66.21) | **(37.65)** | (0.014) | **(0.011)** |
|  | *0.969* | *0.299* | *0.975* | ***0.075*** | *0.809* | ***0.008*** |
| *Left Intraparietal Sulcus* | -11.53 | 100.68 | -17.66 | 31.61 | 0.015 | 0.015 |
|  | (108.31) | (100.16) | (49.31) | (47.16) | (0.013) | (0.012) |
|  | *0.969* | *0.597* | *0.975* | *0.899* | *0.706* | *0.398* |
| *Right Angular Gyrus* | -276.36 | 245.98 | -28.73 | -0.72 | -0.040 | 0.056 |
|  | (160.2) | (116.11) | (38.44) | (30.73) | (0.030) | (0.026) |
|  | *0.507* | *0.205* | *0.975* | *0.981* | *0.706* | *0.186* |
| *Left Angular Gyrus* | -182.89 | 115.76 | 19.1 | 48.19 | -0.059 | -0.039 |
|  | (186.17) | (145.32) | (48.5) | (28.79) | (0.030) | (0.028) |
|  | *0.860* | *0.666* | *0.975* | *0.424* | *0.706* | *0.398* |

Estimates (B), standard deviations (SD) and q-values (q) for the linear associations between all surface-based neocortical measures (volume, surface area and thickness) and RRBs as assessed by the ADI-R, while controlling for autistic symptoms from the social domain, as assessed by the ADI-R. A positive estimate indicates an increase in brain estimate related to more repetitive behavior symptoms. Bold text indicates significant associations (*FDR-corrected q-value* <0.05) or associations with q-value < 0.10 (FDR-corrected). All results are FDR corrected.

**Table S8B.** Within pair model for RRBI’s (from ADI-R) predicting atomy of subcortical and cerebellar ROI’s, while controlling for social interaction deficits (from ADI-R).

| **RRBIs with Social Interaction Control (ADI-R)**  **“Subcortical”** | **SubCortical Volume** | | | | |
| --- | --- | --- | --- | --- | --- |
| ROI's Subcortical | Males B (SD) q | Females B (SD) q | ROI's Subcortical | Males B (SD) q | Females B (SD) q |
| *L. Thalamus Proper* | 51.09 | -34.86 | *L. Cerebellum White* | 29.45 | -85.58 |
|  | (74.53) | (49.42) |  | (197.67) | (106.43) |
|  | *0.704* | *0.687* |  | *0.882* | *0.857* |
| *L. Caudate* | 49.77 | -42.63 | *L. Cerebellum Cortex* | **-1198.49** | -173.12 |
|  | (46.38) | (37.41) |  | **(528.03)** | (281.04) |
|  | *0.704* | *0.540* |  | ***0.070*** | *0.857* |
| *L. Putamen* | -9.18 | -25.12 | *R. Cerebellum White* | -34.92 | -127.79 |
|  | (38.62) | (25.29) |  | (128.23) | (123.78) |
|  | *0.892* | *0.540* |  | *0.882* | *0.857* |
| *L. Pallidum* | 14.58 | -13.55 | *R. Cerebellum Cortex* | **-1092.29** | -180.34 |
|  | (13.04) | (11.48) |  | **(358.74)** | (333.06) |
|  | *0.704* | *0.540* |  | ***0.014*** | *0.857* |
| *L. Amygdala* | 9.67 | 9.27 | *Somato Motor* | 14.15 | 2.41 |
|  | (13.49) | (23.7) |  | (8.79) | (13.34) |
|  | *0.704* | *0.773* |  | *0.215* | *0.857* |
| *R. Thalamus Proper* | 42.48 | -53.67 | *Somato Motor White* | 14.23 | 2.47 |
|  | (51.7) | (31.33) |  | (13.33) | (12.05) |
|  | *0.704* | *0.540* |  | *0.429* | *0.857* |
| *R. Caudate* | 7.4 | -19.64 |  |  |  |
|  | (54.31) | (35.37) |  |  |  |
|  | *0.892* | *0.724* |  |  |  |
| *R. Putamen* | 65.04 | -25.74 |  |  |  |
|  | (53.44) | (22.98) |  |  |  |
|  | *0.704* | *0.540* |  |  |  |
| *R. Pallidum* | 25.11 | -5.64 |  |  |  |
|  | (16.98) | (20.39) |  |  |  |
|  | *0.704* | *0.782* |  |  |  |
| *R. Amygdala* | 3.47 | -16.17 |  |  |  |
|  | (23.39) | (16.4) |  |  |  |
|  | *0.892* | *0.540* |  |  |  |

Estimates (B), standard deviations (SD) and q-values (q) for the within-pair associations between subcortical measures (volumes only) and RRBs as assessed by the ADI-R, while controlling for autistic symptoms from the social domain, as assessed by the ADI-R. A positive estimate indicates an increase in brain estimate related to more repetitive behavior symptoms. Bold text indicates significant associations (*FDR-corrected q-value* <0.05) or associations with q-value < 0.10 (FDR-corrected). All results are FDR corrected. L. = Left, R. = Right.

**Table S9A.** Within-pair model for RRBI’s (from SRS) predicting atomy of neocortical ROI’s, while controlling for social cognition deficits (from SRS).

| **RRBI’s (SRS) with Social Cognition**  **Control (SRS)**  **“Neocortical”** | **Cortical Volume** | | **Surface Area** | | **Cortical thickness** | |
| --- | --- | --- | --- | --- | --- | --- |
|  | Males β (SD) *q* | Females β (SD) *q* | Males β (SD) *q* | Females β (SD) *q* | Males β (SD) *q* | Females β (SD) *q* |
| *Right Lateral Orbital Sulcus* | -12.12 | -4.21 | -2.48 | -2.86 | -0.009 | 0.013 |
|  | (6.19) | (6.35) | (3.57) | (3.45) | (0.007) | (0.008) |
|  | *0.259* | *0.752* | *0.818* | *0.697* | *0.995* | *0.495* |
| *Left Lateral Orbital Sulcus* | 5.05 | -14.84 | 1.63 | **-8.13** | 0.006 | 0.002 |
|  | (7.65) | (6.06) | (3.04) | **(2.05)** | (0.009) | (0.007) |
|  | *0.873* | *0.259* | *0.818* | ***0.003*** | *0.995* | *0.945* |
| *Right Middle Frontal Sulcus* | 44.06 | -17.03 | 17.77 | 3.66 | 0.003 | **-0.011** |
|  | (34.24) | (35.18) | (11.44) | (11.23) | (0.006) | **(0.004)** |
|  | *0.763* | *0.780* | *0.527* | *0.863* | *0.995* | ***0.067*** |
| *Left Middle Frontal Sulcus* | 0.09 | 0.65 | 3.89 | -2.12 | -0.004 | **-0.009** |
|  | (19.16) | (25.21) | (6.56) | (8.39) | (0.006) | **(0.004)** |
|  | *0.996* | *0.980* | *0.818* | *0.871* | *0.995* | ***0.071*** |
| *Right Orbital Gyrus* | 50.73 | -18.75 | 9.32 | -0.82 | 0.006 | -0.002 |
|  | (23.14) | (15.4) | (6.42) | (4.24) | (0.004) | (0.007) |
|  | *0.204* | *0.599* | *0.527* | *0.871* | *0.995* | *0.945* |
| *Left Orbital Gyrus* | 29.77 | -46.1 | 1.09 | -4.47 | 0.008 | -0.004 |
|  | (23.85) | (22.35) | (5.82) | (5.35) | (0.007) | (0.010) |
|  | *0.763* | *0.469* | *0.901* | *0.697* | *0.995* | *0.945* |
| *Right Middle Frontal Gyrus* | 79.06 | 3.12 | 13.26 | 5.99 | 0.002 | -0.006 |
|  | (75.44) | (47.5) | (17.48) | (14.65) | (0.004) | (0.005) |
|  | *0.765* | *0.980* | *0.818* | *0.863* | *0.995* | *0.601* |
| *Left Middle Frontal Gyrus* | 74.64 | -99.83 | 24.39 | -20.75 | -0.007 | -0.006 |
|  | (72.45) | (66.51) | (15.11) | (18.84) | (0.005) | (0.003) |
|  | *0.765* | *0.520* | *0.527* | *0.609* | *0.995* | *0.440* |
| *Right Inferior Frontal Orbital Gyrus* | 18.78 | -1 | 3.18 | -0.87 | 0.0002 | 0.002 |
|  | (8.10) | (17.49) | (1.72) | (2.93) | (0.010) | (0.008) |
|  | *0.204* | *0.980* | *0.436* | *0.863* | *0.995* | *0.9450* |
| *Left Inferior Frontal Orbital Gyrus* | 4.12 | -7.67 | 1.37 | -2.31 | -0.003 | -0.0008 |
|  | (10.54) | (9.84) | (2.58) | (2.48) | (0.011) | (0.008) |
|  | *0.960* | *0.683* | *0.818* | *0.697* | *0.995* | *0.952* |
| *Right Anterior Cingulate Cortex* | 22.83 | 20.5 | 7.54 | 12.15 | -0.002 | -0.006 |
|  | (25.41) | (32.01) | (10.4) | (10.13) | (0.007) | (0.005) |
|  | *0.765* | *0.752* | *0.818* | *0.609* | *0.995* | *0.601* |
| *Left Anterior Cingulate Cortex* | 50.5 | 17.45 | 12.98 | 9.76 | -0.0008 | -0.007 |
|  | (24.8) | (18.84) | (8.84) | (6.22) | (0.003) | (0.007) |
|  | *0.250* | *0.638* | *0.527* | *0.467* | *0.995* | *0.601* |
| *Right PostCentral Gyrus* | -40.13 | -41.96 | -16.22 | 1.64 | 0.0004 | **-0.017** |
|  | (25.37) | (25.34) | (8.32) | (7.81) | (0.008) | **(0.006)** |
|  | *0.512* | *0.520* | *0.436* | *0.871* | *0.995* | ***0.056*** |
| *Left PostCentral Gyrus* | 3.34 | 33.77 | 8.25 | 12.24 | -0.007 | -0.0002 |
|  | (20.5) | (30.17) | (6.03) | (7.45) | (0.006) | (0.007) |
|  | *0.979* | *0.599* | *0.560* | *0.452* | *0.995* | *0.977* |
| *Right PostCentral Sulcus* | -21.07 | -1.95 | -8.03 | -0.93 | -0.0003 | -0.006 |
|  | (46.25) | (23.63) | (23.81) | (10.62) | (0.004) | (0.004) |
|  | *0.934* | *0.980* | *0.874* | *0.930* | *0.995* | *0.558* |
| *Left Post Central Sulcus* | -7.37 | -5.69 | 3.69 | 5.95 | -0.006 | -0.001 |
|  | (29.09) | (24.01) | (13.00) | (8.37) | (0.004) | (0.005) |
|  | *0.960* | *0.973* | *0.874* | *0.735* | *0.995* | *0.945* |
| *Right PreCentral Gyrus* | -104.66 | 23.94 | **-27.92** | 4.3 | -0.006 | 0.001 |
|  | (38.59) | (25.27) | **(6.83)** | (6.87) | (0.008) | (0.011) |
|  | *0.120* | *0.638* | ***0.002*** | *0.754* | *0.995* | *0.945* |
| *Left PreCentral Gyrus* | -31.78 | 73.96 | 6.07 | 19.47 | -0.013 | 0.001 |
|  | (37.34) | (25.55) | (7.79) | (9.23) | (0.008) | (0.005) |
|  | *0.765* | *0.136* | *0.818* | *0.210* | *0.995* | *0.945* |
| *Right Inferior PreCentral Sulcus* | -23.92 | 24.74 | -11.64 | 11.75 | -0.005 | -0.006 |
|  | (32.26) | (19.66) | (11.07) | (5.98) | (0.007) | (0.005) |
|  | *0.825* | *0.599* | *0.818* | *0.254* | *0.995* | *0.558* |
| *Left Inferior PreCentral Sulcus* | 53.72 | 12.42 | 16.48 | 7.82 | 0.002 | -0.008 |
|  | (24.11) | (21.7) | (8.52) | (9.12) | (0.005) | (0.006) |
|  | *0.204* | *0.780* | *0.436* | *0.697* | *0.995* | *0.558* |
| *Right Superior PreCentral Sulcus* | -8.08 | -29.23 | -2.05 | -16.35 | -0.007 | -0.002 |
|  | (16.02) | (28.67) | (6.78) | (13.85) | (0.006) | (0.009) |
|  | *0.934* | *0.616* | *0.874* | *0.609* | *0.995* | *0.945* |
| *Left Superior PreCentral Sulcus* | -8.35 | 23.75 | -0.33 | 10.39 | -0.010 | -0.004 |
|  | (17.79) | (22.85) | (7.49) | (9.41) | (0.005) | (0.007) |
|  | *0.934* | *0.616* | *0.965* | *0.609* | *0.995* | *0.873* |
| *Right Central Sulcus* | -61.23 | 6.86 | **-32.08** | 4.07 | 0.001 | -0.003 |
|  | (20.94) | (13.59) | **(10.25)** | (6.72) | (0.004) | (0.003) |
|  | *0.120* | *0.780* | ***0.032*** | *0.754* | *0.995* | *0.601* |
| *Left Central Sulcus* | -4.26 | 38.45 | -3.52 | 15.78 | -0.0003 | 0.002 |
|  | (13.7) | (20.14) | (6.16) | (11.27) | (0.003) | (0.003) |
|  | *0.960* | *0.474* | *0.818* | *0.485* | *0.995* | *0.784* |
| *Right Superior Frontal Sulcus* | -28.81 | -26.13 | -11.01 | -6.77 | 0.003 | -0.003 |
|  | (34.18) | (30.33) | (11.72) | (9.80) | (0.004) | (0.005) |
|  | *0.765* | *0.667* | *0.818* | *0.735* | *0.995* | *0.784* |
| *Left Superior Frontal Sulcus* | 5.38 | -32.42 | 2.83 | -6.12 | -0.004 | -0.007 |
|  | (31.85) | (41.02) | (11.47) | (11.85) | (0.004) | (0.004) |
|  | *0.979* | *0.683* | *0.878* | *0.808* | *0.995* | *0.495* |
| *Right Superior Frontal Gyrus* | -71.89 | -58.75 | -10.23 | -3.92 | 2e-05 | **-0.009** |
|  | (86.09) | (40.25) | (20.24) | (11.44) | (0.003) | **(0.003)** |
|  | *0.765* | *0.520* | *0.818* | *0.863* | *0.995* | ***0.055*** |
| *Left Superior Frontal Gyrus* | -62.48 | 26.01 | -10.62 | 19.53 | 0.0005 | -0.005 |
|  | (55.28) | (53.6) | (12.75) | (13.81) | (0.004) | (0.004) |
|  | *0.765* | *0.780* | *0.818* | *0.485* | *0.995* | *0.558* |
| *Right Supramarginal Gyrus* | -3.71 | 87.69 | -12.16 | **33.04** | 0.012 | -0.002 |
|  | (50.56) | (47.67) | (16.11) | **(12.25)** | (0.009) | (0.012) |
|  | *0.996* | *0.474* | *0.818* | ***0.063*** | *0.995* | *0.945* |
| *Left Supramarginal Gyrus* | -17.37 | -79.35 | -6.86 | -16.91 | 0.004 | -0.002 |
|  | (65.68) | (57.77) | (23.45) | (19.07) | (0.009) | (0.010) |
|  | *0.960* | *0.555* | *0.874* | *0.697* | *0.995* | *0.945* |
| *Right Superior Parietal Gyrus* | -1.31 | 60.89 | -0.67 | **26.44** | 0.002 | -0.007 |
|  | (44.76) | (41.64) | (10.17) | **(10.16)** | (0.008) | (0.005) |
|  | *0.996* | *0.520* | *0.965* | ***0.066*** | *0.995* | *0.558* |
| *Left Superior Parietal Gyrus* | -2.6 | 8.62 | 10.82 | -4.07 | -0.005 | 0.007 |
|  | (32.1) | (42.22) | (14.78) | (11.81) | (0.009) | (0.006) |
|  | *0.996* | *0.973* | *0.818* | *0.863* | *0.995* | *0.558* |
| *Right Intraparietal Sulcus* | 11.38 | 6.88 | 7.57 | 13.11 | 0.004 | -0.005 |
|  | (40.91) | (41.29) | (16.32) | (18.27) | (0.004) | (0.004) |
|  | *0.960* | *0.976* | *0.826* | *0.735* | *0.995* | *0.558* |
| *Left Intraparietal Sulcus* | -13.15 | -37.05 | -7.02 | -20.93 | 0.002 | 0.003 |
|  | (26.06) | (33.32) | (12.43) | (14.15) | (0.004) | (0.003) |
|  | *0.934* | *0.599* | *0.818* | *0.485* | *0.995* | *0.602* |
| *Right Angular Gyrus* | 67.92 | -75.04 | 11.81 | **-31.05** | 0.003 | 0.006 |
|  | (80.61) | (46.26) | (15.46) | **(10.4)** | (0.008) | (0.011) |
|  | *0.765* | *0.520* | *0.818* | ***0.051*** | *0.995* | *0.878* |
| *Left Angular Gyrus* | 69.32 | -41.83 | 20.8 | **-33.04** | 0.003 | **0.023** |
|  | (62.32) | (36.86) | (11.59) | **(11.64)** | (0.011) | **(0.007)** |
|  | *0.765* | *0.599* | *0.436* | ***0.054*** | *0.995* | ***0.018*** |

Estimates (B), standard deviations (SD) and q-values (q) for the within pair associations between all surface-based neocortical measures (volume, surface area and thickness) and RRBs as assessed by the SRS, while controlling for autistic symptoms from the social domain, as assessed by the SRS. A positive estimate indicates an increase in brain estimate related to more repetitive behavior symptoms. Bold text indicates significant associations (*FDR-corrected q-value* <0.05) or associations with q-value < 0.10 (FDR-corrected). All results are FDR corrected.

**Table S9B.** Within-pair model for RRBI’s (from SRS) predicting atomy of subcortical and cerebellar ROI’s, while controlling for social cognition deficits (from SRS).

| **RRBI’s (SRS) with Social Cognition**  **Control (SRS)**  **“Subcortical”** | **SubCortical Volume** | | | | |
| --- | --- | --- | --- | --- | --- |
| ROI's Subcortical | Males B (SD) q | Females B (SD) q | ROI's Subcortical | Males B (SD) q | Females B (SD) q |
| *L. Thalamus Proper* | 7.97 | -7.77 | *L. Cerebellum White* | 122.52 | 70.85 |
|  | (25.58) | (17.63) |  | (74.61) | (49.88) |
|  | *0.965* | *0.864* |  | *0.302* | *0.508* |
| *L. Caudate* | 7.16 | -15.87 | *L. Cerebellum Cortex* | -123.67 | 29.9 |
|  | (14.37) | (11.02) |  | (179.15) | (102.23) |
|  | *0.965* | *0.467* |  | *0.557* | *0.770* |
| *L. Putamen* | 2.52 | -15.26 | *R. Cerebellum White* | 148.47 | 41.04 |
|  | (10.82) | (11.56) |  | (105.47) | (54.21) |
|  | *0.965* | *0.467* |  | *0.319* | *0.674* |
| *L. Pallidum* | -0.37 | 6.44 | *R. Cerebellum Cortex* | -163.86 | 108.12 |
|  | (4.59) | (8.18) |  | (154.55) | (83.42) |
|  | *0.965* | *0.718* |  | *0.434* | *0.508* |
| *L. Amygdala* | 3.42 | -10.71 | *Somato Motor* | -6.98 | 2.36 |
|  | (5.28) | (5.88) |  | (3.2) | (4.45) |
|  | *0.965* | *0.385* |  | *0.174* | *0.716* |
| *R. Thalamus Proper* | 13.94 | 13.09 | *Somato Motor White* | -2.43 | 6.24 |
|  | (21.08) | (14.78) |  | (4.14) | (5.47) |
|  | *0.965* | *0.718* |  | *0.557* | *0.508* |
| *R. Caudate* | -1.17 | -23.09 |  |  |  |
|  | (13.89) | (13.06) |  |  |  |
|  | *0.965* | *0.385* |  |  |  |
| *R. Putamen* | -15.81 | -1.62 |  |  |  |
|  | (11.34) | (9.44) |  |  |  |
|  | *0.817* | *0.864* |  |  |  |
| *R. Pallidum* | 7.05 | 1.51 |  |  |  |
|  | (4.34) | (5.88) |  |  |  |
|  | *0.817* | *0.864* |  |  |  |
| *R. Amygdala* | -0.29 | 1.58 |  |  |  |
|  | (6.47) | (7.1) |  |  |  |
|  | *0.965* | *0.864* |  |  |  |

Estimates (B), standard deviations (SD) and q-values (q) for the within pair associations between subcortical measures (volumes only) and RRBs as assessed by the SRS, while controlling for autistic symptoms from the social domain, as assessed by the SRS. A positive estimate indicates an increase in brain estimate related to more repetitive behavior symptoms. Bold text indicates significant associations (*FDR-corrected q-value* <0.05) or associations with q-value < 0.10 (FDR-corrected). All results are FDR corrected. L. = Left, R. = Right.

**Table S10A.** Linear model assessing the interaction between age and RRBI symptoms (ADI-R) and neocortical regions of interest.

| **Interaction age X RRBI (ADI-R)**  **“Neocortical”** | **Cortical Volume**  β (SD) q | **Surface Area**  β (SD) q | **Cortical thickness**  β (SD) q |
| --- | --- | --- | --- |
| *Right Lateral Orbital Sulcus* | -1.46 | -0.46 | -0.004 |
|  | (1.78) | (0.96) | (0.002) |
|  | *0.759* | *0.962* | *0.330* |
| *Left Lateral Orbital Sulcus* | -2.24 | -1.52 | 0.003 |
|  | (1.64) | (0.76) | (0.002) |
|  | *0.528* | *0.684* | *0.416* |
| *Right Middle Frontal Sulcus* | 11.65 | 1.94 | 0.002 |
|  | (8.12) | (2.61) | (0.002) |
|  | *0.528* | *0.962* | *0.574* |
| *Left Middle Frontal Sulcus* | 1.19 | -0.39 | -0.001 |
|  | (6.28) | (2.34) | (0.003) |
|  | *0.874* | *0.999* | *0.712* |
| *Right Orbital Gyrus* | -9.48 | -3.48 | 0.002 |
|  | (9.35) | (2.59) | (0.002) |
|  | *0.759* | *0.760* | *0.487* |
| *Left Orbital Gyrus* | -5.27 | -2.99 | 0.002 |
|  | (13.6) | (2.27) | (0.003) |
|  | *0.851* | *0.760* | *0.614* |
| *Right Middle Frontal Gyrus* | 20.29 | 0.06 | 0.003 |
|  | (16.79) | (5.97) | (0.002) |
|  | *0.634* | *0.999* | *0.404* |
| *Left Middle Frontal Gyrus* | 15.26 | 0.84 | 0.002 |
|  | (20.99) | (5.99) | (0.002) |
|  | *0.759* | *0.999* | *0.632* |
| *Right Inferior Frontal Orbital Gyrus* | 2.02 | -0.59 | 0.006 |
|  | (3.65) | (0.83) | (0.003) |
|  | *0.816* | *0.962* | *0.114* |
| *Left Inferior Frontal Orbital Gyrus* | 0.66 | -0.35 | 0.002 |
|  | (2.99) | (0.68) | (0.003) |
|  | *0.874* | *0.962* | *0.637* |
| *Right Anterior Cingulate Cortex* | 3.38 | -0.69 | 0.003 |
|  | (9.05) | (2.97) | (0.002) |
|  | *0.851* | *0.999* | *0.278* |
| *Left Anterior Cingulate Cortex* | 5.69 | 1.08 | 0.002 |
|  | (12.07) | (3.66) | (0.002) |
|  | *0.851* | *0.999* | *0.632* |
| *Right PostCentral Gyrus* | **24.33** | 4.24 | 0.004 |
|  | **(6.55)** | (2.21) | (0.002) |
|  | ***0*** | *0.684* | *0.278* |
| *Left PostCentral Gyrus* | 1.49 | -3.04 | 0.004 |
|  | (6.73) | (2.62) | (0.003) |
|  | *0.874* | *0.858* | *0.390* |
| *Right PostCentral Sulcus* | 17.04 | 4.46 | 0.005 |
|  | (8.45) | (3.23) | (0.002) |
|  | *0.276* | *0.760* | *0.114* |
| *Left Post Central Sulcus* | -3.97 | -3.29 | 0.001 |
|  | (10.15) | (5.28) | (0.002) |
|  | *0.851* | *0.962* | *0.637* |
| *Right PreCentral Gyrus* | -6.11 | -0.51 | -0.001 |
|  | (11.27) | (2.79) | (0.003) |
|  | *0.816* | *0.999* | *0.830* |
| *Left PreCentral Gyrus* | -2.68 | -0.5 | 0 |
|  | (12.42) | (3.44) | (0.004) |
|  | *0.874* | *0.999* | *0.908* |
| *Right Inferior PreCentral Sulcus* | -6.19 | -1.28 | -0.002 |
|  | (6.97) | (2.75) | (0.002) |
|  | *0.759* | *0.9615* | *0.390* |
| *Left Inferior PreCentral Sulcus* | 6 | 0.14 | 0.002 |
|  | (6.99) | (2.42) | (0.001) |
|  | *0.759* | *0.999* | *0.390* |
| *Right Superior PreCentral Sulcus* | **-16.36** | -7.06 | 0.001 |
|  | **(4.8)** | (2.32) | (0.002) |
|  | ***0.018*** | *0.108* | *0.799* |
| *Left Superior PreCentral Sulcus* | 1.67 | -0.64 | 0.001 |
|  | (5.95) | (2.4) | (0.001) |
|  | *0.874* | *0.999* | *0.637* |
| *Right Central Sulcus* | 4.82 | 0.17 | 0.001 |
|  | (6.7) | (3.14) | (0.002) |
|  | *0.759* | *0.999* | *0.632* |
| *Left Central Sulcus* | 0.41 | -2.15 | 0.001 |
|  | (3.72) | (2.9) | (0.002) |
|  | *0.913* | *0.962* | *0.637* |
| *Right Superior Frontal Sulcus* | 12.27 | -0.25 | 0.005 |
|  | (17.83) | (6.47) | (0.002) |
|  | *0.759* | *0.999* | *0.114* |
| *Left Superior Frontal Sulcus* | -15.05 | -5.09 | -0.001 |
|  | (10.84) | (4.89) | (0.001) |
|  | *0.528* | *0.900* | *0.637* |
| *Right Superior Frontal Gyrus* | 12.59 | -3.2 | 0.003 |
|  | (16.72) | (6.11) | (0.002) |
|  | *0.759* | *0.962* | *0.266* |
| *Left Superior Frontal Gyrus* | -15.05 | -11.41 | 0.004 |
|  | (22.59) | (6.92) | (0.002) |
|  | *0.759* | *0.727* | *0.114* |
| *Right Supramarginal Gyrus* | 29.1 | 3.1 | 0.007 |
|  | (14.44) | (5.13) | (0.005) |
|  | *0.276* | *0.962* | *0.383* |
| *Left Supramarginal Gyrus* | 12.17 | -3.09 | 0.008 |
|  | (17.93) | (4.37) | (0.004) |
|  | *0.759* | *0.962* | *0.266* |
| *Right Superior Parietal Gyrus* | 22.59 | 0 | **0.006** |
|  | (14.37) | (3.8) | **(0.002)** |
|  | *0.472* | *0.999* | ***0.036*** |
| *Left Superior Parietal Gyrus* | 41.15 | 7.17 | 0.003 |
|  | (15.51) | (4.84) | (0.003) |
|  | *0.108* | *0.760* | *0.416* |
| *Right Intraparietal Sulcus* | -13.23 | -9.55 | 0.002 |
|  | (13.43) | (5.75) | (0.002) |
|  | *0.759* | *0.727* | *0.349* |
| *Left Intraparietal Sulcus* | 17.92 | 4.94 | 0.002 |
|  | (8.91) | (4.39) | (0.002) |
|  | *0.276* | *0.858* | *0.550* |
| *Right Angular Gyrus* | 30.58 | -3.72 | 0.009 |
|  | (19.39) | (5.08) | (0.004) |
|  | *0.472* | *0.962* | *0.114* |
| *Left Angular Gyrus* | 27.1 | -1.37 | 0.008 |
|  | (14.29) | (2.9) | (0.005) |
|  | *0.309* | *0.962* | *0.278* |

Estimates (B), standard deviations (SD) and q-values (q) for the linear interaction associations between age (years) and RRBIs as assessed by the ADI-R on neocortical volume, surface area and thickness. Bold text indicates significant associations (*FDR-corrected q-value* <0.05) or associations with q-value < 0.10 (FDR-corrected). All results are FDR corrected.

**Table S10B.** Linear model assessing the interaction between age and RRBI symptoms (ADI-R) and subcortical regions of interest.

| **Interaction age X RRBI (ADI-R)**  **“Subcortical”** | **SubCortical Volume** | | |
| --- | --- | --- | --- |
| ROI's Subcortical | B (SD) q | ROI's Subcortical | B (SD) q |
| *L. Thalamus Proper* | -0.75 | *L. Cerebellum White* | -9.52 |
|  | (8.59) |  | (16.91) |
|  | *0.930* |  | *0.754* |
| *L. Caudate* | -3.7 | *L. Cerebellum Cortex* | 47.99 |
|  | (5.84) |  | (77.58) |
|  | *0.803* |  | *0.754* |
| *L. Putamen* | 6 | *R. Cerebellum White* | -12.47 |
|  | (6.87) |  | (17.93) |
|  | *0.766* |  | *0.754* |
| *L. Pallidum* | 1.13 | *R. Cerebellum Cortex* | 31.48 |
|  | (1.94) |  | (71.65) |
|  | *0.803* |  | *0.754* |
| *L. Amygdala* | **6.07** | *Somato Motor* | 1.60 |
|  | **(2.18)** |  | (1.62) |
|  | ***0.060*** |  | *0.754* |
| *R. Thalamus Proper* | 7.95 | *Somato Motor White* | -0.50 |
|  | (5.42) |  | (1.59) |
|  | *0.360* |  | *0.754* |
| *R. Caudate* | -1.74 |  |  |
|  | (6.1) |  |  |
|  | *0.930* |  |  |
| *R. Putamen* | 13.24 |  |  |
|  | (7.77) |  |  |
|  | *0.300* |  |  |
| *R. Pallidum* | 0.49 |  |  |
|  | (2.41) |  |  |
|  | *0.930* |  |  |
| *R. Amygdala* | 4.71 |  |  |
|  | (2.41) |  |  |
|  | *0.265* |  |  |

Estimates (B), standard deviations (SD) and q-values (q) for the linear interaction associations between age (years) and RRBIs as assessed by the ADI-R on subcortical volumes. Bold text indicates significant associations (*FDR-corrected q-value* <0.05) or associations with q-value < 0.10 (FDR-corrected). All results are FDR corrected. L. = Left, R. = Right.
